# Consolidation of metabolomic, proteomic, and GWAS data in connective model of schizophrenia

**DOI:** 10.1038/s41598-023-29117-7

**Published:** 2023-02-06

**Authors:** Arthur T. Kopylov, Alexander A. Stepanov, Tatiana V. Butkova, Kristina A. Malsagova, Natalia V. Zakharova, Georgy P. Kostyuk, Artem U. Elmuratov, Anna L. Kaysheva

**Affiliations:** 1 Biobank Group, Department of Proteomic Research, Institute of Biomedical Chemistry, 10 Pogodinskaya Str., Bld. 8, Moscow, Russian Federation 119121; 2 Alexeev N.A. 1St Clinics for Mental Health, 2 Zagorodnoe Road, Moscow, Russian Federation 115119; 3 Center for Medical Genetics “Genotek”, 17/1 Nastavnichesky Lane, Moscow, Russian Federation 105120

**Keywords:** Synaptic plasticity, Transporters in the nervous system, Steroid hormones, Thyroid hormones, Neurochemistry, Modularity, Systems analysis, Schizophrenia

## Abstract

Despite of multiple systematic studies of schizophrenia based on proteomics, metabolomics, and genome-wide significant loci, reconstruction of underlying mechanism is still a challenging task. Combination of the advanced data for quantitative proteomics, metabolomics, and genome-wide association study (GWAS) can enhance the current fundamental knowledge about molecular pathogenesis of schizophrenia. In this study, we utilized quantitative proteomic and metabolomic assay, and high throughput genotyping for the GWAS study. We identified 20 differently expressed proteins that were validated on an independent cohort of patients with schizophrenia, including ALS, A1AG1, PEDF, VTDB, CERU, APOB, APOH, FASN, GPX3, etc. and almost half of them are new for schizophrenia. The metabolomic survey revealed 18 group-specific compounds, most of which were the part of transformation of tyrosine and steroids with the prevalence to androgens (androsterone sulfate, thyroliberin, thyroxine, dihydrotestosterone, androstenedione, cholesterol sulfate, metanephrine, dopaquinone, etc.). The GWAS assay mostly failed to reveal significantly associated loci therefore 52 loci with the smoothened *p* < 10^−5^ were fractionally integrated into proteome-metabolome data. We integrated three omics layers and powered them by the quantitative analysis to propose a map of molecular events associated with schizophrenia psychopathology. The resulting interplay between different molecular layers emphasizes a strict implication of lipids transport, oxidative stress, imbalance in steroidogenesis and associated impartments of thyroid hormones as key interconnected nodes essential for understanding of how the regulation of distinct metabolic axis is achieved and what happens in the conditioned proteome and metabolome to produce a schizophrenia-specific pattern.

## Introduction

Schizophrenia is fundamentally a mental disorder main feature of which is focused on psychotic symptoms and articulated cognitive impairments. Investigation of schizophrenia as a separate disorder started in 1896 from the researches of Emil Kraepelin’s who enrolled cognate attributes such as catatonia, hebephrenia, chronic delirium, and early dementia into a single nosology which he called as “*dementia praecox*”. A few years later Eugen Bleuler criticized the hypothesis about paramount importance of the early dementia assuming that the integrating characteristics for the observed disorder are dissociating in cognitive function and significant perceptual alterations that demonstrate excessive flexibility. This has been termed as “*schizophrenia*” in 1911.

The burden of responsibility for the correct diagnosis of schizophrenia and fetching the net efficiency of multidrug therapy are still challenging tasks^1,2^. The high complexity of schizophrenia may delude even expertized clinicians because of there is extended range of symptoms that may overlap with other mental illnesses^3^. Therefore, accurate diagnosis of schizophrenia and its distinction are yet accomplished in support with NCS-R or MMPI survey and DSM-5 criteria applied to persons who are suspected of having mental or variety of related issues^4,5^.

Psychotic symptoms (delusions and bizarre behavior) are the most emphasized in onset and during schizophrenia^4,6^. These are generally accounted as secondary consequences while there are variety evidence that perceptual and cognitive disturbances are pivotal attributes of the illness since they manifest even in the absence of pronounced psychotic symptoms. In this respect, schizophrenia can be confessed as a neurodegenerative brain disorder^7^. However, if the risk to be affected by neurodegenerative disease is increased dramatically with an age, the onset of schizophrenia is typically emerging between late teens and up to thirties years old^6,7^.

Our understanding of schizophrenia remains mostly incomplete due to elusive interplay between different factors, including genetic, social, environmental, and difficulties in generating accurate animal models for the assessment of underlying mechanisms^6^. Relevant markers with reliable prognostic and diagnostic values are ultimately required. The sufficient opportunity may provide clinical proteomic investigations with a satisfying history of the discovery of tentative biomarkers. Although there are limitations that are defined by standardization and heterogeneity in the study design, yet several promising markers are repeatedly recognized in proteomics of psychopathologies.

Recent reviews for mental illnesses established a satisfied overlap across a few dozen proteomic investigations^7^. Most of the listed and affected during schizophrenia proteins are related to lipids metabolism, complement system, and PPAR/RXR signaling^8^. The cohort of HP, SERPINA1, CFB, APOA1, APOA4, APOA2, APOC, TTR, APCS, and AMBP is typically found as affected proteins^7,9,10^. The conception of the immune response to endogenous stimuli suspects interleukins (IL-1ra, IL-8, IL-10, IL-15, IL-16, IL-17, and IL-18) as meaningfully varied in schizophrenia patients even at the first episode^8,9^. Some reports attended the oxidative stress, considering the pronounced alterations in PRDX, GSTM3, and NADPH-dependent oxidoreductases^8,11^.

The main objection raised is the specificity of the observed biomarkers. Most of the serum-originated proteins are typically overrepresented; thence, the shortcoming is their redundancy among as neurodegenerative disorders, including Parkinson’s and Alzheimer’s diseases^12,13^ as in variety of unrelated disorders. It seems on the surface that such circumstance erodes the diagnostic value at cost of low selectivity and specificity^13^. Thence, numerous of proteomic markers are placed in a detrimental position, but better position can be achieved through the combination with metabolomics and GWAS.

Schizophrenia is an extraordinary disease with yet too uncertain molecular mechanism. Hence, as far complex assay targets the disease, as many limitations follow the study. Our study is observational and bears several limitations, of which small size of population (dimensionality) is only one of them. Apparently, the resulting GWAS was unsatisfied due to insufficient size of study and validating cohorts. This make sense but we had to go through this hurdle to display consistency of the previously observed GWAS results with proteomic and metabolomic data which are less sensitive to study population size. The prevalence of the observed metabolic and proteomic compounds, and candidates of loci in this study, are established intentionally. Nevertheless, we are reluctant to recognized a comprehensive model of schizophrenia pathogenesis and to find patterns because such elaboration requires strict correlations and strong statistics on a significantly larger size of study population. Yet as that seems, complex and individual scheme of therapy of patients with schizophrenia is another hurdle that hardly to overcome because vast medication greatly contributes in metabolic background and shadows metabolic transformations relevant to the pathology of a verity. We convinced that enrichment with quantitative data, associated GWAS, and metabolic compounds found in the same patients, may disclose the clue signaling pathways and multipotent molecular events.

## Results

### Population

**Figure 1:**
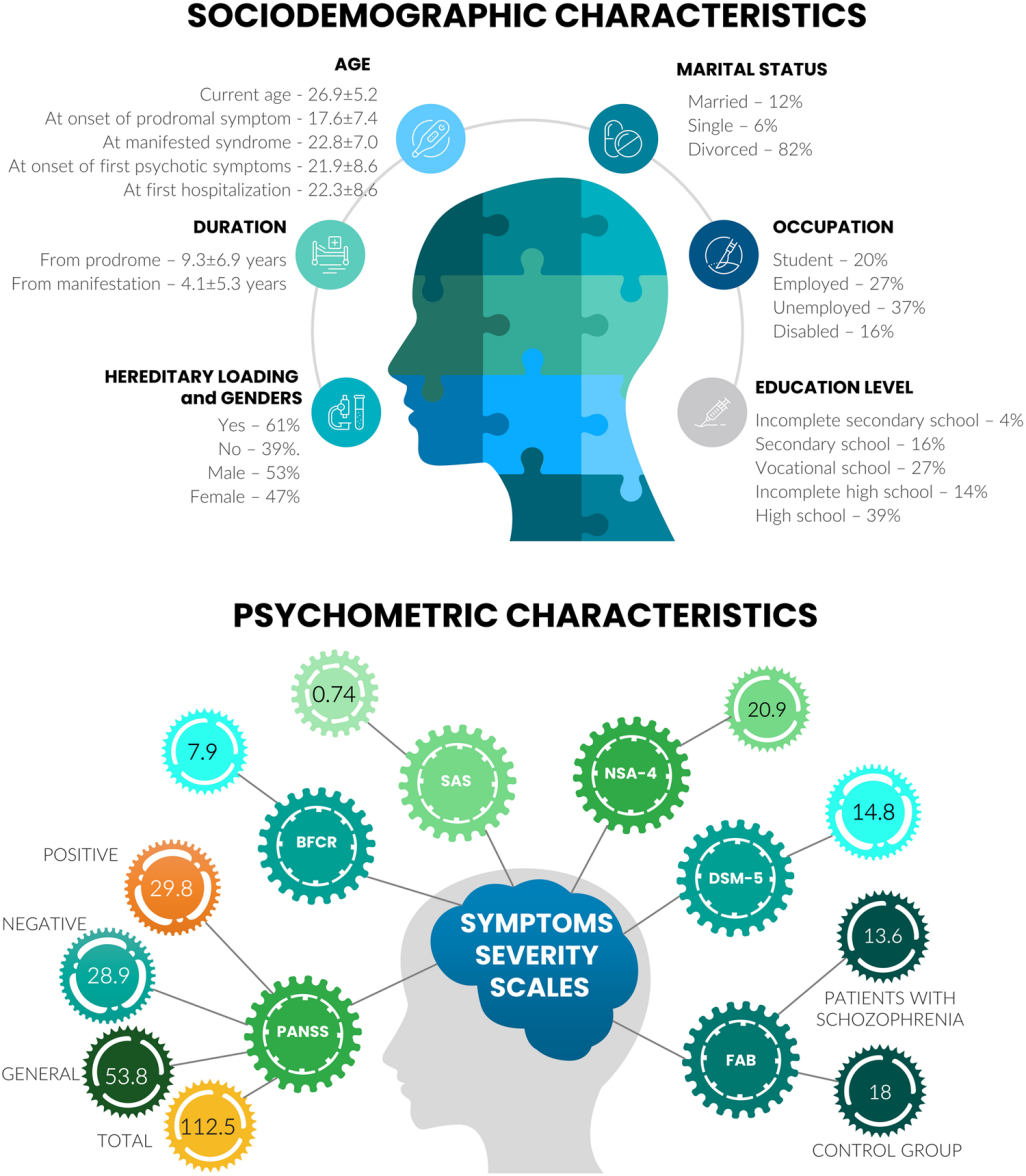
Main anthropometric and psychometric data accrued for subjects with schizophrenia (n = 49). Complete records can be appreciated in the Supplementary Appendix A (Demography) and include the corresponding characteristics for the control group of healthy donors (n = 50) and for the validating group of patients with schizophrenia (n = 28). All groups under consideration were aligned by age and genders ratio items. Both, study population and validating group were leveled in the duration from prodrome and the duration from manifestation. The hereditary loading was slightly inclined in the study cohort (61%) cmpare to the validating group (46%; see Supplementary Appendix A) however this did not affect the final results and conclusions. Subjects of study cohorts were tested with several scales to assess the severity and progression of symptoms (Mann–Whitney test at a raw p < 0.05): *PANSS* Positive and Negative Syndrome Scale, *BFCR scale* Bush–Francis Catatonia Rating Scale, *NCS4* the 4-Item Negative Symptom Assessment, *SAS* Simpson-Angus Scale, *DSM-5* Diagnostic and Statistical Manual of mental disorders, fifth edition, *FAB* Frontal Assessment Battery.

We examined 77 patients with schizophrenia, including 28 subjects from validating cohort, and 61 healthy donors, including 11 subjects from validating cohort (Fig. 1) The prevalence of schizophrenic subjects in the consolidated (study and validating cohorts) are characterized by heredity loading (61%). Patients were treated by drug therapy to target the underlying cognitive dysfunction and negative symptom dimension of full-blown schizophrenia, or attenuated psychosis syndrome and specific endophenotypes related to the increased risk of psychosis. The enrolled population of patients favored to equal distribution of psychometric scores for positive symptoms (e.g., hallucinations and thought disorders which are the core of the disease) and negative symptoms (e.g., flat affect and social withdrawal) but most were characterized by severe cognitive (e.g., learning and attention disorders) symptoms (Fig. 1).

Monitoring of clinical symptoms and evaluation of proteome, metabolome, and GWAS were conducted for patients who undergo inpatient treatment initiated with one or more of the following antipsychotic drugs: chlorpromazine (intravenously), olanzapine (tablets), biperiden (tablets), clozapine (tablets), risperidone, lithium carbonate (tablets), ziprasidone (tablets), elzepam (tablets), haloperidol (tablets). In most cases (82%) risperidone in dose of 6.7–8 mg/day was selected as the main antipsychotic drug, and the rest of patients were treated by olanzapine (15–20 mg/day) or zuclopenthixol (28 mg/day), and clozapine was chosen for augmentation of antipsychotic agent.

Clinical and neurological symptoms were rated using standard rating scales (Fig. 1, Supplementary Appendix A) and the efficiency of multidrug therapy was evaluated as lytic (gradual) reduction of symptoms severity for 76% of patients after 17–24 weeks of therapy. The prolonged, i.e., more than 8 weeks, persistence of acute symptoms was recognized for 16% of patients with recommendation of simultaneous use of two or more incisive antipsychotics with high doses; and the rest of patients (8%) are characterized by a cease of seizure during the first 2 weeks after initiation of therapy.

### Serological markers

**Figure 2:**
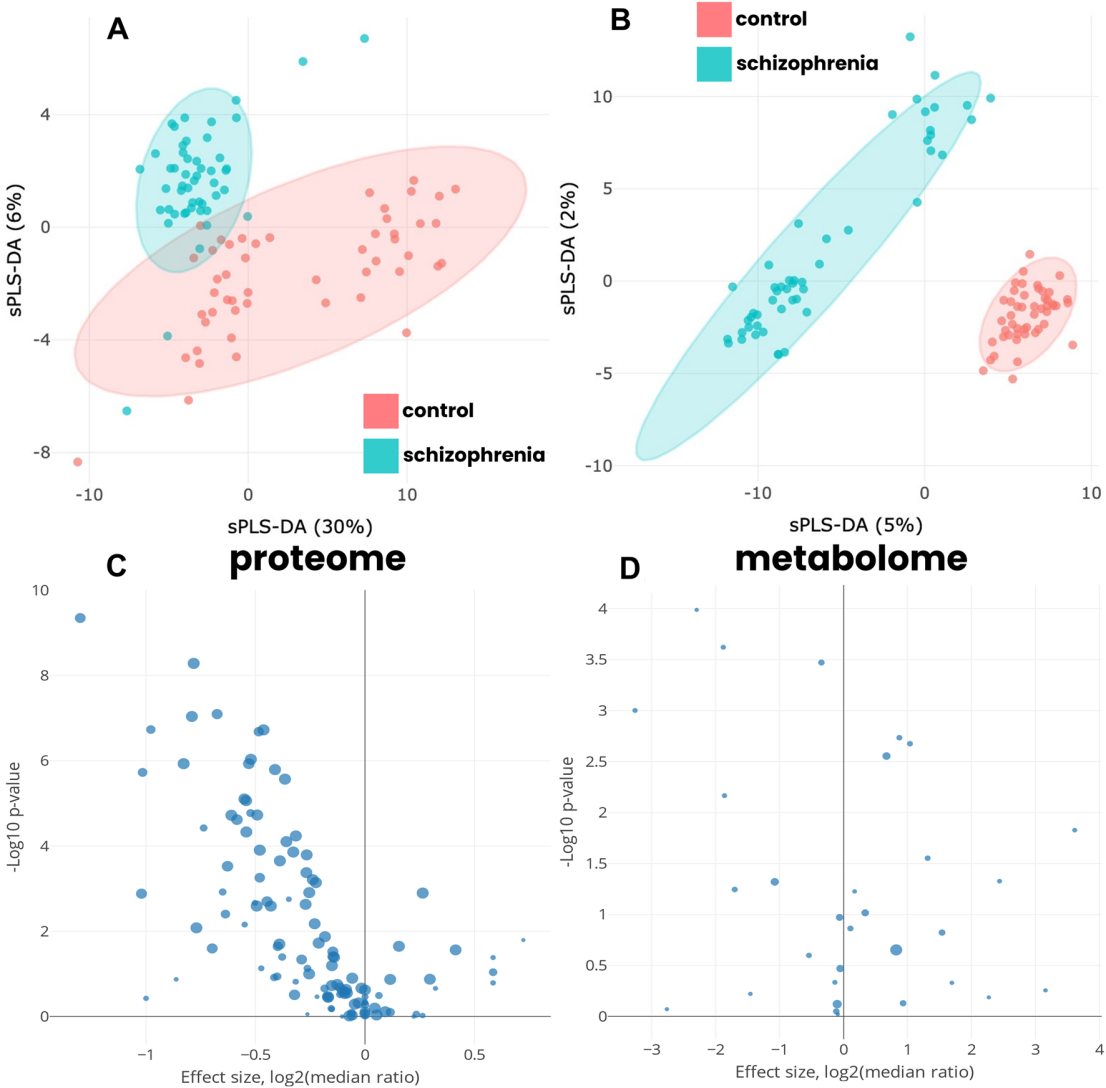
Discriminant analysis of studied cohorts and scatter volcano plot of the most significantly altered proteins and metabolites. Sparce partial least-squares discriminant analysis (sPLS-DA) for proteome (**A**) and metabolome (**B**) data type with 0.95 ellipse confidence level. The designed score scattering plots show relationship between the control group and patients with schizophrenia, and the degree of variations that were explained by each component consisted of PC1 = 30% and PC2 = 6% for proteomic data (**A**) and of PC1 = 5% and PC2 = 2% for metabolomic data (**B**). A volcano plot for the most significant variables determined in proteome (**C**) and in metabolome (**D**) that enabled to discriminate the control group from patients with schizophrenia (Supplementary Appendix C, Appendix D). Only proteins and metabolites with scores exceeding FC = 2 (in linear scale) and p-value above 0.05 (Mann–Whitney U-test) were considered as significant and were engaged for the reconstruction of multilayer molecular events chain. All other analyses were performed with the in-house scripts written in R (version 3.2.0; R Foundation for Statistical Computing, Vienna, Austria; https://www.r-project.org/).

Totally n = 250 different proteins were identified, among which n = 217 were specified for the control group, whereas n = 199 proteins were attributed to SCZ patients. If the identified peptide was not undoubtedly linked to a specific protein isoform, all isoforms were grouped and classified as one protein. Principal components analysis (PCA) failed to separate groups of study and the majority of subjects are densely overlapped after examination of the complete proteome and metabolomic profiles (Supplementary Appendix A). Therefore, to achieve discrimination, we performed variable selection and classification using sparce partial least-squares discriminant analysis (sPLS-DA), which displayed successful segregation of subjects with schizophrenia from healthy donors with 0.95 ellipse area confidence level on the layer of proteome (Fig. 2A) and metabolome (Fig. 2B). The resulting discriminant analysis explained variability of 30% and 6% for the consolidated proteome (Fig. 2A), and 5% and 2% for the consolidated metabolome (Fig. 2B).

Among significantly differed proteins, APOC3 (p = 0.000696), PLMN (p = 0.000232), APOH (p = 0.006), A1AG1 (p = 0.002), VTDB (p = 0.015), APOB (p = 0.001), ITIH4 (p = 0.011), and ITIH1 (p = 0.001) were the most meaningful elements. In majority, these proteins belong to lipids metabolism (including cholesterol uptake and dependent steroidogenesis) and associated synthesis and transport of ligands acting in chronic inflammatory reaction. The presence of two ITIH isoforms (ITIH1 and ITIH4) among differently expressed proteins supports the ongoing process of extracellular matrix remodeling, which is general at inflammation and morphogenic re-arrangements of cytoarchitecture. Both processes are typically determined in patients with resistant schizophrenia especially in entorhinal cortex^14^.

The abundance of members of complement system reflects the sensitivity to morphological changes in brain cortex since re-activation of complement factors contributes greatly in neurodegenerative disorders and in brain morphology and architecture in schizophrenic patients^15^. However, pairwise t-test did not reveal significant differences for C8A/B/G chains (*p* = 0.182), C4BP (*p* = 0.086), and CFAB (*p* = 0.615), but significance has been found for C1R (*p* = 0.00321), C1QC (*p* = 0.008), and C3 (*p* = 0.01) members of the complement system. Apparently, the immune system is flexible and sensitive but it is adaptive to background stimuli, which biases the net input of complement system in pathogenesis unless dynamic monitoring of the disease history is performed. Nevertheless, the disclosed pattern assumingly indicates the immune reactivity in the classical pathway, instead of alternative pathway, which is profoundly dysregulated in schizophrenia^16^.

When conducted contrast comparison of studied groups, 159 overlapped proteins (Fig. 2C) were established (Supplementary Appendix C). To extract the shared proteins, initial semi-quantitative analysis, and protein filtering were run as indicated in Supplementary Appendix B and C. Annotation of biological processes in terms of GO supported the incline to negative regulation of re-modelling LDL (*p* = 9.17e−06), positive regulation of substrate-dependent cells migration (*p* = 5.54e−04), Kinin–Kallikrein cascade activation (*p* = 5.96e−04), acute inflammatory response (*p* = 1.04e−20), positive regulation of humoral immune response (*p* = 2.26e−29), positive regulation of complement alternative pathway (*p* = 6.62e−16), and response to oxidative stress (*p* = 1.22e−03).

Regarding the cellular localization, most of determinants were assigned to extracellular matrix (*n* = 134; *p* = 3.07e−12), cell periphery/membrane proteins (*n* = 87, *p* = 5.71e−04) and secretory vesicles (*n* = 116, *p* = 7.04e−12). An overwhelming majority of proteins belong to multiple (overlapping) localizations, which is caused by close neighborhood of the interplaying processes. The contrast analysis based on the semi-quantitative indicator (NSAF) between study groups returned *n* = 24 proteins with *p* < 0.01 (Fig. 2C). Among the established proteins, four (16.7%) were downregulated, and twenty (83.3%) were upregulated (Table 1) but four proteins (GPX3, IGKC, C2 and ATRN) did not pass the significance score when running the validating cohort albeit they were credibly quantified in the training cohort (Supplementary Appendix C).

**Table 1:** Most significantly altered proteins measured in the study cohort (estimated as fold-changes; 1 < log2FC < (− 1) in logarithmic scale, Benjamini–Hochberg adjusted *p*-value cut-off *p* < 0.01) and in the validation cohort (estimated in absolute concentration at adjusted *p*-value cut-off *p* < 0.01) of patients with schizophrenia.

Primary gene name	Pathway description (Reactome identifier)	Study cohort, SCZ patients		Validating cohort			
		Fold changes	Adjusted *p*-value	Concentration, mean ± SD, nM/L		Fold changes	Adjusted *p*-value*
				SCZ group	Control group		
IGFALS	Growth hormone synthesis, secretion and action (hsa04935)	− 1.47	0.063	0.073 ± 0.280	0.551 ± 0.432	− 2.92	7.73E−08
ORM1	–	− 0.36	2.81E−06	30.09 ± 11.57	43.00 ± 15.04	− 0.52	5.49E−05
AHSG	–	1.53	7.43E−05	89.66 ± 33.12	25.43 ± 6.490	1.82	1.14E−11
PLG	Neuroactive ligand-receptor interaction (hsa04080); Complement and coagulation cascades (hsa04610)	1.41	1.16E−06	57.34 ± 19.09	15.31 ± 2.701	1.91	6.62E−13
SERPINF1	Wnt signaling pathway (hsa04310)	1.65	0.0017	1.741 ± 1.210	0.433 ± 0.440	2.00	2.34E−06
ITIH4	–	2.27	0.0002	39.25 ± 9.181	8.218 ± 1.561	2.25	7.12E−18
C8A	Complement and coagulation cascades (hsa04610); Corovirus disease—COVID-19 (hsa05171)	1.64	0.0026	1.712 ± 1.740	0.354 ± 0.412	2.27	2.16E−04
A1BG	–	1.55	1.56E−05	47.03 ± 15.92	9.244 ± 2.273	2.35	1.00E−13
SERPINF2	Complement and coagulation cascades (hsa04610)	2.49	2.95E−07	9.644 ± 3.594	1.799 ± 0.951	2.42	5.12E−13
SERPIND1		1.62	0.0005	9.222 ± 3.441	1.675 ± 1.082	2.46	2.66E−13
SERPINA1		3.48	0.0001	649.1 ± 178.9	113.3 ± 26.83	2.52	2.23E−16
GC	Vitamin digestion and absorption (hsa04977); Cholesterol metabolism (hsa04979);	1.32	8.60E−05	136.7 ± 32.65	22.95 ± 4.917	2.58	3.53E−18
CP	Ferroptosis (hsa04216)	2.31	7.08E−05	89.32 ± 22.87	14.44 ± 2.684	2.63	2.33E−17
GSN	Regulation of actin cytoskeleton (hsa04810)	3.36	6.23E−05	13.00 ± 3.662	1.880 ± 0.584	2.79	1.40E−16
APOB	Vitamin digestion and absorption (hsa04977); Cholesterol metabolism (hsa04979); Fat digestion and absorption (hsa04975); Lipid and atherosclerosis (hsa05417)	2.52	1.57E−05	158.1 ± 55.38	21.74 ± 7.380	2.86	4.05E−14
VTN	Complement and coagulation cascades (hsa04610); ECM-receptor interaction (hsa04512); Focal adhesion (hsa04510); PI3K-Akt signaling pathway (hsa04151)	2.54	1.72E−05	25.07 ± 9.713	3.420 ± 1.205	2.87	5.03E−13
APOH	Cholesterol metabolism (hsa04979)	2.61	2.76E−05	60.40 ± 18.59	7.243 ± 3.247	3.06	6.73E−16
ITIH1	Fluid shear stress and atherosclerosis (hsa05418); Glycosaminoglycan degradation (hsa00531)	2.52	1.74E−06	36.43 ± 12.07	3.846 ± 1.121	3.24	4.29E−15
APOC3	Cholesterol metabolism (hsa04979); PPAR signaling pathway (hsa03320)	2.53	9.37E−07	33.12 ± 18.69	2.639 ± 2.424	3.65	7.49E−10
IGKV3-20	–	5.62	3.81E−07	4.325 ± 3.833	0.007 ± 0.050	9.23	9.74E−07

Absolute concentrations in *nmols* per a liter (nM) were estimated by support of the plotted calibration curve of the UPS-2 standard. Significance was accepted if measured concentration exceeded double the standard deviation and Benjamini–Hochberg adjusted *p*-value cut-off of *p* < 0.01. Proteins GPX3, IGKC, C2 and ATRN did not pass the validation filters (Supplementary Appendix C). Proteins are ordered according to their abundancy and concentration (FC, fold changes in logarithmic scale) in the validating cohort of patients.

Proteins that did not pass statistical validation (p < 0.01) but featured by a high biological significance.

*p-value adjusted by the Benjamini–Hochberg correction for multiple testing.

### Quantification of serological protein markers

The selected proteins (*n* = 24 proteins) were tested as an integrated panel against the distinct (secondary) validation cohort of SCZ patients (*n* = 28) and subjects of the secondary control group (*n* = 11) to confirm preliminary results. The secondary cohort of SCZ patients was enrolled from the subjects aligned with the train group in sociodemographic and psychometric characterization. The definitive collection of significantly altered proteins was quantified using the approximation of LFQ (Label-Free Quantification) values plotted against the known concentrations of 48 different proteins of the UPS-2 (Universal Proteome Standard Dynamic Range 10^–5^–10^–12^ M) spiked into the matrix and acquired in six independent replicates within a range of 4 orders of magnitude (Supplementary Appendix B). The estimated concentrations in SCZ patients varied between 0.026 and 649 nmol/L, while the baseline in the control group ranged between 0.007 and 203 nmol/L (Table 1). It should be noticed, that the concentrations the upper measured limit is more than 3-folds higher in subjects with schizophrenia.

Previous studies reported that the total proteins fraction (in plasma) is decreased in schizophrenics compared to healthy donors, however the disproportion can be tracked in the selected plasma protein fractions. In particular, α1-fraction increased (FC = 2.1, *p* < 0.001) in patients with severe symptoms whereas α2-fraction and β-fraction were decreased^17^. Some studies associated the increase of α2-fraction with the high psychomotor activity of schizophrenia subjects, but reports about α1-fraction are ambiguous and controversial^18^. In our study some proteins of α1-fraction were definitely decreased (A1AT; Table 1), which is inconsistent with the previously reported data, however we did not report about other proteins (for example, transcortin and thyroid-binding globulin) of α1-fraction, which are differed insignificantly compared to the control group (Supplementary Appendix C). Being as a part of α2-fraction, CERU was found among significantly decreased proteins in the group of schizophrenia, which falls with the finding of elevation of α2-fraction at acute forms of schizophrenia and decreased solubility of plasma proteins recognized in brains of such patients^19^. It should also be admitted that among proteins significantly differed in quantitative measure, GPX3, IGKC, C2, and ATRN (adjusted *p* < 0.01) did not pass the significance level in the validation cohort of subjects with schizophrenia due to large deviation (see Supplementary Appendix C).

### Functional annotation

Protein–protein interactions (PPI) for the selected and validated *n* = 20 proteins (Table 1) were reconstructed using the Search Tool for Retrieval of Interacting Genes/Proteins (STRING). Among the quantified proteins that increase in SCZ patients, 11 were implicated (in GO terms) in the regulation of hydrolase activity (GO:0051336), and 13 were attributed to the response to stress (GO:0006950) with the local cluster coefficients of 0.775 (PPI enrichment *p* = 2.28e−06 and *p* = 0.00022, respectively; Supplementary Appendix C). At the same time, the majority of proteins (*n* = 14) were involved in the regulation of inflammatory response (GO:0050727, *p* = 0.00062) and *n* = 11 proteins related to the regulation of response to a stimulus (GO:0048583, *p* = 0.0118). Both decreasing proteins (ALS and A1AG1; Table 1, Supplementary Appendix C) fit to the process of response to stress, and A1AG1 also displays participation in vesicle-mediated transport (GO:0016192, *p* = 2.71e−05). The determined cellular components for the majority of quantified proteins (Supplementary Appendix C), as expected, were extracellular space and secretion (87.5%, GO:0005576, *p* = 6.14e−16) or localization in the intermembrane systems (58.3%; GO:0012505, *p* = 0.00026). The prevalent molecular functions designated for the quantified proteins were enzyme regulatory activity including inhibitor activity (*n* = 9 proteins; GO:0030234, *p* = 1.10e−05), heparin binding (*n* = 4 proteins; GO:0008201, *p* = 0.0004), steroid and lipids binding (*n* = 6 proteins; GO:0005496 and GO:0008289, *p* = 0.0353 and *p* = 0.0242).

Analysis of molecular reactions and signaling pathways extracted from the KEGG and Reactome databases for the listed proteins exhibited the predominance of regulation of the complement cascade and the immune system (HSA-977606 and HSA-166658, *p* = 4.93e−34), regulation of hemostasis (HSA-109582, *p* = 4.40e−23), post-translational proteins modification (HSA-597592, *p* = 0.00034), extracellular matrix organization (HSA-1474244, *p* = 0.00079), regulation, assembly, remodeling and clearance of LDL and VLDL particles (HSA-8964041, HSA-8866423, HSA-8964046 and HSA-8963896 with *p* = 0.0028, *p* = 0.0038, *p* = 0.0047 and *p* = 0.0059, correspondingly).

### Metabolomic markers

The untargeted approach determined 1159 and 1573 metabolites amongst healthy donors and SCZ patients, correspondingly. Suggesting that metabolomic data can be largely ambiguous, we wondered about the validation through the permutation test to observe the inflation of errors incrementing false-positive results. Eventually, we excluded 226 insignificant compounds, most of which inferred with drug-like, non-endogenous and non-human metabolites.

**Figure 3:**
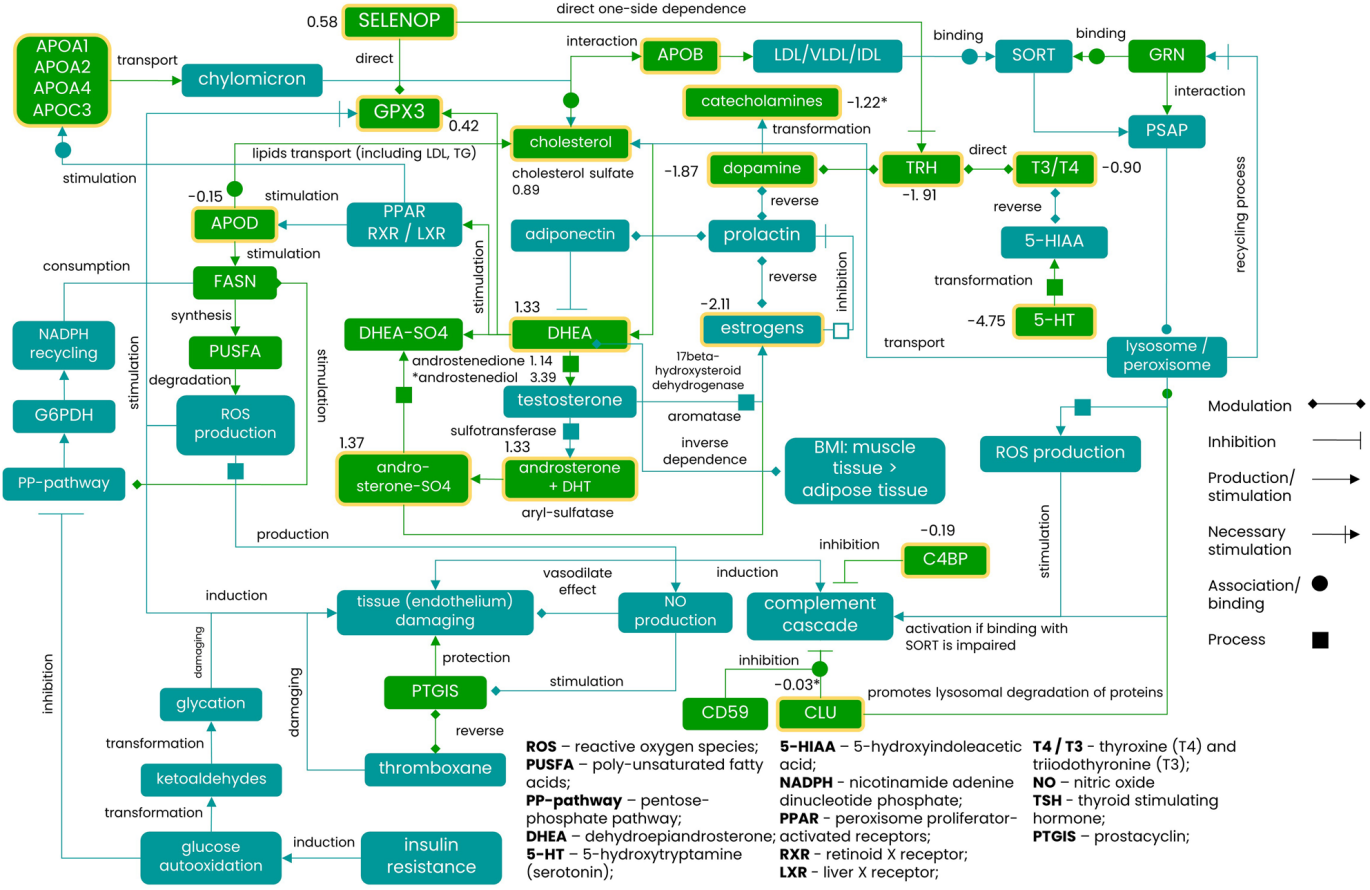
The proposed integrative scheme of interplay between proteome and metabolome layers in patients with schizophrenia. Observed elements (metabolites and proteins) are highlighted by yellow frame and FC (fold-changes) value; the FC observed but insignificant (after running the validation cohort of patients with schizophrenia) are designated by asterisk (*). Although it is almost impossible to figure out the very initial point, still there are several biological processes explicitly outlined on the proposed plane. It seems that the immune response and lipids metabolism might be one of the initial points affected in schizophrenia. The declination of APOA2, APOA4 and APOC1 and lipocalins APOE, APOM, APOH, CLU in combination with the increased oxidative-induced factors (GPX3, APOD, SELENOP) might be a clue. It is worth noticed that the consolidation of apolipoproteins with action of FASN, GPX3 and PEDF in steroidogenesis produces significant influence on the neuroimmune HPA (hypothalamic–pituitary–adrenal) axis. By this reason, some steroids, including DHEA and precursors of 17-ketosteroids, and the balance between androgen and estrogen (sex hormones) pathways bears a special significance. The concept of DHEA role was considered as important in pathogenesis of schizophrenia even before due to pro-oxidative and defending properties initiated through affecting on PPARα/γ1/γ2 receptors and, thus, regulation of apolipoproteins family genes expression. The connecting point is enhanced by the expression of ROS-sensitive APOD that triggers FASN and, consequently, enhance ROS generation. It entails to initiation of complement cascade as a response on the local inflammatory, where some complement factors (C5, C3, and C2 complement factors) enhance cAMP production through the Ca^2+^-dependent signaling. In turn, it boosts excessive NO production which readily interacts with ROS species and aggravates immune response closing the loop with cAMP. This, in sense, may found a response in the action of PTGIS prostaglandin. The progenitor guiding gene PTHG was consistently found in the studied subjects as associated loci and it is perfectly match with the hypothesized escalation of inflammation/immune response and steroidogenesis stressed through cAMP and Ca^2+^-dependent signaling. Another sort of neuroimmune regulation undergoes through the axis of prolactin and its surrounding. Here, the comprehensive and complementary interplay between prolactin and 17β-estradiol, and opposed impact on adiponectin and dopamine are crucial. Apart direct regulation of steroidogenesis, it impacts on the transformations of tyrosine including its transformation products dopamine, dopaquinone, and catecholamines, which has been observed imbalanced. As an obvious consequence, TRH and thyroid hormones tend to decrease which is agreed with the obtained data and previously reported observations about hypothyroidism in the affected patients.

After contrast analysis that credibly meet criteria of identification (Supplementary Appendix B), we focused on 142 common metabolites (Supplementary Appendix D). As for proteomic serological markers, sPLS-DAdiscrimination achieved better dispersion between the control and SCZ groups (Fig. 2B). Pairwise comparison (Benjamini–Hochberg adjusted *p* < 0.005) did not reveal significant features if handling the complete metabolic profiles of studied groups. However, among the shared part of metabolome, a dozen (*n* = 12) of metabolites were characterized with the highest difference between the control group and patients with schizophrenia (*U*-test, adjusted *p* < 0.05; Table 2). These metabolites were incorporated into the reconstructing map of molecular events to exhibit substantial differences between the baseline and SCZ patients (Fig. 3).

**Table 2:** The most significantly altered metabolites (estimated as fold-changes; 1 < log_2_FC < (− 1) in logarithmic scale, *p*-value cut-off *p* < 0.05) associated with proteins observed and measured in proteome layer in the study cohort and in the independent validating cohort of patient with schizophrenia.

Name of metabolite	Pathway description	Main associated identifiers		Study cohort		Validating cohort	
		KEGG ID	HMDB ID	Fold changes	Adjusted *p*-value^†^	Fold changes	Adjusted* p*-value^†^
Serotonin	Neuroactive ligand-receptor interaction (hsa04080); Tryptophan metabolism (hsa00380); cAMP signaling pathway (hsa04024); Synaptic vesicle cycle (hsao4721); Serotonergic synapse (hsa04726); Bile secretion (hsa04976); Chemical carcinogenesis—receptor activation (hsa05207	C00780	HMDB00259	− 3.26	1.00E−03	− 4.75	0.002
Thyrotropin-releasing hormone	Tyrosine metabolism (hsa00350); Thyroid hormone synthesis (hsa04918)	C03958	–	− 2.3	1.00E−04	− 1.91	0.007
3-O-Sulfogalactosylceramide	Sphingolipid metabolism (hsa00600)	C06125	HMDB00024	− 1.88	2.00E−04	− 1.73	0.037
Estrone	Steroid hormone biosynthesis (hsa00140); Ovarian steroidogenesis (hsa04913); Prolactin signaling pathway (hsa04917)	C00468	HMDB00145	− 1.86	6.80E−03	− 2.11	0.258
Thyroxine	Tyrosine metabolism (hsa00350); Thyroid hormone synthesis (hsa04918)	C08212	HMDB00248	− 1.08	4.79E−02	− 0.90	0.035
L-3,5-Diiodotyrosine	Tyrosine metabolism (hsa00350); Thyroid hormone synthesis (hsa04918)	C01060	–	− 0.35	3.00E−04	− 0.18	0.034
Cholesterol sulfate	Steroid hormone biosynthesis (has00140)	C18043	HMDB00653	0.67	2.80E−03	0.89	0.040
16-a-Hydroxypregnenolone	Lipid metabolism pathway (has01212); Steroid hormone biosynthesis (has00140)	C06390	HMDB00315	0.87	1.80E−03	1.06	0.006
Dihydrotestosterone	Steroid hormone biosynthesis (has00140)	C03917	HMDB02961	1.04	2.10E−03	1.33	0.002
Androstenedione	Steroid hormone biosynthesis (has00140); Ovarian steroidogenesis (has04913); Prolactin signaling pathway (has04917)	C00280	HMDB00053	1.31	2.81E−02	1.14	0.165
Vitamin D3 (7-dehydrocholesterol)	Steroid biosynthesis (has00100); Vitamin digestion and absorption (has04977); Rheumatoid arthritis (has05323)	C05443	–	2.44	4.72E−02	5.02	0.021
Androsterone sulfate	Steroid hormone biosynthesis (hsa00140)	C04555	HMDB02759	3.61	1.49E−02	1.37	0.025

The observed metabolites are ordered according to their representation (fold changes in logarithmic (log_2_ scale) estimated toward the corresponding control groups. In overwhelming majority, identified metabolites are part of tyrosine/catecholamines pathway and transformation of steroids with incline to androgens and secosteroids.

^†^p-value calculated for the validating cohort at a significance cut-off of *p* < 0.05 with Benjamini–Hochberg correction.

The volcano plot (Fig. 2D) highlights metabolites with FDR *p* < 0.05 and fold changes more than two and less than 0.5. The collected metabolome comprised of steroids and amines transformation products, thyroid-related hormones, derivatives and esters of cholesterol, amino acids, sphingosines and ceramide (Table 2). Six metabolites (metanephrine, norepinephrine, dopamine, sphingosine, dopaquinone, androstanediol) are with substantial biological meaning but did not pass the statistical significance and left the main list of metabolites while were running through the validating cohort (Supplementary Appendix D) despite their prevalence and the high frequency (more than 0.8) of quantitative loading among study groups.

Since two cohorts (assay group of patients and validating group of patients) were aligned by anthropometric and psychometric parameters, this partial inconsistence can be caused by the dimensionality effect, i.e., sample size. Interestingly, testosterone (T; *p* = 0.407) and its conjugates (sulfate and glucuronide) did not fit the final dataset of promising metabolites in either study and validating groups, however we expected an opposite result. Changes in sex hormones, especially, in testosterone are abundantly discussed in the context of schizophrenia. Admittedly, the frequency of schizophrenia incidences is almost twice higher among males and they experience more severe negative and cognitive symptoms. While the onset is especially achieved from the beginning of adolescent age^20^, it is also tightly associated with hyperprolactinemia. However, other researchers reported that the disturbance of testosterone level is correlated with the medication treatment strategy, since unmedicated patients (of them are mostly patients with the first episode) demonstrated the normal level of testosterone^21,22^, but the higher level of DHEA^23^. At the same time, there many reports favoring the “estrogen” theory and suggesting that female sex hormones can play a protective role against schizophrenia even by promoting the synaptic plasticity. Thence, the incidence rate of schizophrenia, mood and depression among females are characterized by the second peak nearly menopause age^24,25^ due to the age-related estrogen deficiency or secondary deficiency caused by hyperprolactinemia. Below, our study indicates that the estrogen hypothesis is more advanced at least in sustainability of schizophrenia symptomatology.

### Candidates within associated loci

The analysis used a soft threshold for MAF. We used the filter C_U ≥ 10 and C_A ≥ 10 and the analysis of GWAS results, excluding the related and overlapping samples, did not identify significant polymorphisms (*p* = 10^−5^) that would distinguish the studied cohorts. Hence, polymorphisms closest to the cut-off threshold (top-100) were nominated, 52 of which were localized in protein-coding regions and considered as an auxiliary for the reconstructing of vulnerable processes in the cross-layer molecular analysis but enhanced by numerous credible GWAS studies. Summary of population structure of common variant (MAF higher than 5%) was tested using a PCA analysis showed that population is homogenous enough but still there was a small fraction of cases that did overlap the control (Supplementary Appendix E). In order to use only cases matching the control samples and to ameliorate population stratification in the GWAS analysis, all cases that did not fall into the area delimited by the mean and three standard deviations of the two first principal components of the control samples were excluded from further consideration. The results are summarized in Supplementary Appendix E but herein we only refer to the relevant associated loci with no significant scores and attempt to link the obtained results with those credible that have been previously established in relevant publications.

At least 20 meaningful biological processes were associated with the identified polymorphisms and can be coated on the protein and metabolite layers. These includes fat digestion and absorption (hsa04975), vitamin digestion (hsa04977), metabolism of cholesterol (hsa04979) and arachidonic acid (hsa00590), viral carcinogenesis (hsa05203), and neuroactive ligand-receptor interaction (hsa04080), etc., in proteome layers; and processes exacerbated in alcoholism (hsa05034), signaling mediated by cAMP (hsa04024) serotonin mediated transduction (hsa04726), sensory taste transduction (hsa04742) and bile secretion process (hsa04976), etc. in metabolome layer (Table 3).

**Table 3:** The relationship between proteomic, metabolomic, and GWAS layers through biological processes in KEGG terms.

Pathway KEGG ID	Pathway description	Proteome: primary gene name	GWAS: SNP ID	Metabolome: KEGG ID*
hsa00590	Arachidonic acid metabolism	GPX3	rs8046 (PTGS1), rs10306166 (PTGS1)	–
hsa04024	cAMP signaling pathway	–	rs118125840 (GABBR2)	C00547, C00780, C03758
hsa04071	Sphingolipid signaling pathway	–	rs10908703 (FCER1A)	C00319
hsa04080	Neuroactive ligand-receptor interaction	PLG	rs2299542 (GRM8), rs118125840 (GABBR2)	C00547, C00780, C03758
hsa04151	PI3K-Akt signaling pathway	VTN	rs2179158 (TNR)	–
hsa04310	Wnt signaling pathway	SERPINF1	rs60428975 (SMAD3)	–
hsa04510	Focal adhesion	VTN	rs2179158 (TNR)	–
hsa04512	ECM-receptor interaction	VTN	rs2179158 (TNR)	–
hsa04726	Serotonergic synapse	–	rs8046 (PTGS1), rs10306166 (PTGS1)	C00780
hsa04913	Ovarian steroidogenesis	–	rs61941687 (SCARB1)	C00280, C00468
hsa04915	Estrogen signaling pathway	GABBR2	rs118125840 (SCARB1)	–
hsa04918	Thyroid hormone synthesis	GPX3		C01060
hsa04923	Regulation of lipolysis in adipocytes	–	rs8046 (PTGS1), rs10306166 (PTGS1)	C00547
hsa04975	Fat digestion and absorption	APOB	rs61941687 (SCARB1)	–
hsa04976	Bile secretion	–	rs61941687 (SCARB1)	C00780, C03758, C04555
hsa04977	Vitamin digestion and absorption	APOB	rs11254275 (CUBN), rs61941687 (SCARB1)	C05443
hsa04979	Cholesterol metabolism	APOH, APOB, APOC3	rs61941687 (SCARB1)	–

*Refer to Table 2 for Metabolite KEGG IDs definition.

## Discussion

The common feature of all proteomic and metabolomic studies regardless the specimen (brain tissue or plasma sample) is alterations observed in energy metabolism, including glycolysis and mitochondria functioning, lipids transformation, and oxidative stress, which integrates changes in various other energy metabolism pathways. In this study, numerous of proteomic and metabolomic elements being involved in pathways touched above have also been found and integrated into the proposed map of schizophrenia and probably they shape the central core of impaired biological processes. The collected proteins, metabolites and putatively associated loci cover 68 biological processes totally, among which only two pathways (hsa04080: Neuroactive ligand-receptor interaction; and hsa04977: Vitamin digestion and absorption) are characterized by intersection of all three layers (Table 3) but include functionally unrelated proteins and metabolites. Some associations are extraordinary relevant to the major hypothesis of the onset and etiology of schizophrenia, including serotonin and dopamine as significantly altered compounds (Table 2), and associated gene polymorphisms (*GRM8*, *PTGS1*, *GABBR2*), covering glutamatergic, serotonergic, GABAergic, and dopaminergic synapse transmission and. Parallel transcriptomic and metabolomic study of post-mortem prefrontal cortex endorses the toxic effect of aberrant glutamatergic transmission on synaptic plasticity and myelinization in patients with schizophrenia^26^. It should be notices, that the *PTGS1* loci was characterized by two distinct SNPs (rs8046 and rs10306166; Table 3) and the resulting protein product is a part of serotonergic and glutamatergic synapse plasticity and transmission (hsa04726; Table 3).

### Energy metabolism and lipids metabolism as constants at schizophrenia

The review of proteomes among psychopathology researches indicates only a small fraction of proteins (A2M, APOA1, APOB, APOC1, APOH, C4BPA, etc.) reasonable to schizophrenia^27^. Evidence exists about relationship with various defensins and interleukins (IL-1ra, IL-8, IL-10, IL-15, etc.), which are differentially expressed at schizophrenia^28,29^. Most of promising protein markers were detected in this study and joined with the established metabolic compounds (Fig. 3). The most challenging issue is that some of these proteins are also constantly proposed for Parkinson’s and Alzheimer’s diseases that exhibits their weak specificity^30,31^. Notwithstanding, due to the majority of studies highlights lipids metabolism as generally observed affected pathway at schizophrenia, we assume that its relationship with the cholesterol metabolism (hsa04979) and associated signaling is the most essential reference point due to the interplay between APOM, carrying 9-*cis*-retinoic acid ligand, and APOD, affecting the RXR/PPAR receptors; both regulate the expression of numerous of apolipoproteins (Fig. 3).

Violation of lipid metabolism and shift in redox balance towards the production of reactive oxygen species (ROS) strongly correlates with APOD regulation^32,33^. Synthesis and secretion of APOD are inversely dependent on the concentration and duration of exposure to 17β-estradiol^34^. Therefore, combinatory therapy of antipsychotic drugs with estradiol shows a positive effect on the recovery of patients with schizophrenia due to targeted action on the expression of receptors and transporters of serotonin in the forebrain and due to the decrease of APOD, which is associated with prolactin antagonizing the estrogens production^35^.

Negative symptoms of schizophrenia are curtained by the complex disturbance of lipids metabolism, oxidative stress, and pro-inflammatory reaction at the prodromal period. We found that the combination of apolipoproteins (APOA2, APOA4, and APOC1) and nonclassical lipocalins (APOE, APOD, APOM, APOH, CLU) tends to moderate declination (Table 1). Several studies reported the predictive power of these apolipoproteins for highly specific differentiation of schizophrenia with specificity above 83%^9,36^. However, data about the APOE implication are the most ambiguous and contradictive. While many studies review the importance of APOE in organization of glia cytoarchitecture and synapse plasticity, considering APOE polymorphisms as candidates for risk of schizophrenia^37^, the meta-analysis of 28 studies revealed such association only in Asian population^38^. In this study, we did not detect APOE as a significantly altered protein, however other members (APOH, APOB, APOC3) of cholesterol metabolism and PPAR signaling were differed compared to the control group (Table 1).

Since we did not find sensitive SNPs of the detected apolipoproteins but the level of these proteins is markedly increased in patients with schizophrenia (Table 1), assumingly other mechanisms implicated in apolipoproteins signal transduction and receptor-mediated binding might be more tightly associated with the disease. The GWAS defined associated SNP (rs61941687) only for *SCARB1* gene (Supplementary Appendix E) related to hsa04979 and hsa04975 (Table 3). Despite *SCARB1* is a receptor of lipophilic ligands, including phospholipids, there are no credible data about relation of exactly *SCARB1* to schizophrenia, albeit changing of phospholipids profile was established in metabolomic assay of patients with schizophrenia and bipolar disorder. However, such changes were found in patients after treatment by lithium and risperidone^39,40^, which were also used in treatment strategy of our study. Probably, a few detectable in the study phospholipids remained unaltered due to post-processes cleaning of metabolic data from drug-affected and drug-related metabolites (see Supplementary Appendix D).

### Governors of oxidative stress and immunoreactivity

Ultimately, dysregulation of energy metabolism and lipids metabolism entails to overproduction of reactive oxygen species and immune response. Dysregulation of redox-responsible proteins, including PRDX6, GFAP, MDH and ATPase subunits, have been repeatedly identified in plasma and prefrontal cortex of patients with schizophrenia^41–43^. Unfortunately, researchers pay little attention to mechanisms instructing the pathogenesis of schizophrenia. We found highly abundant GPX3 that indicates an over-scored ROS generation since the enzyme utilizes end-products of GSP and SOD due to increased lipids peroxidation. Earlier, alterations in GST, A2M, and IL-17 were demonstrated for 25%, 17%, and 20% of patients^9^. Assumingly this confirms our suggestion about stressful oxidative damage; thus, signs of experienced apoptosis can be expected, among which BCL9 was undoubtedly distinguished (Supplementary Appendix C).

Closer examination revealed progranulin (GRN) as a potential marker of schizophrenia. It regulates inflammation and transport to lysosomes, and acts as an IL-8 inducer. The association between GRN, neurodegenerative diseases (including dementia), and bipolar disorder has been reliably recognized^44^. At least in murine models, deficiency of GRN provokes autophagy and impaired signaling in neurons^45^. GRN is a secreted protein and regulated by TFEB^46^. The protein is delivered and exported to lysosomes after heterodimerization with prosaposin. Some mutations in GRN induce the increased activity of TFEB and upregulation of lysosome-specific proteins together with hyperactivity of the innate immunity^47^. As a result, neurons and microglia cells include large-size mature lysosomes, which ultimately entails to cell toxicity and rapidly expanding inflammation processes^48^, both are confirmed by the elevated level of CERU, CLU, and complement factors (Table 1).

**Figure 4:**
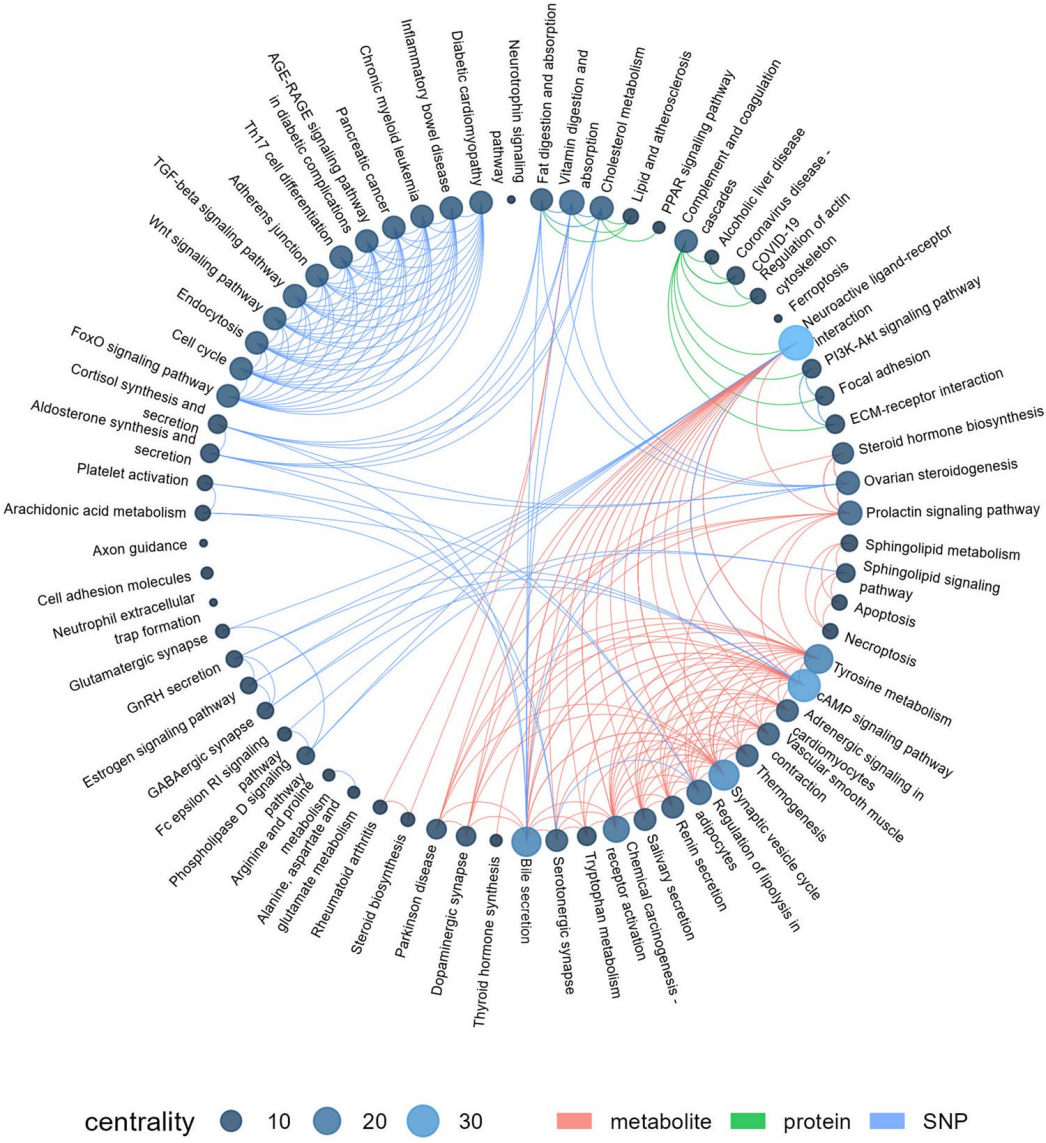
Circular plot for the Multi Omics connections between the serum-based proteome, metabolome and SNPs recognized in patients with schizophrenia. The external environment of the graph shows the most affected biological pathways where the identified meaningful molecular factors (Table 3) are involved in. The graph size was equal to 554 with assortative value of 0.346. Connections (colored lines, or edges) entering and outputting in/from different pathways (nodes, or vertices) illustrate a track caused by common elements between molecular layers (proteome—red line; metabolome—green line; gene with SNP—blue color) that has been defined in the study (Table 3) and consolidated with the current knowledge about pathophysiological mechanism of schizophrenia. If entity (node) has no connection (edge) with other node, there is no element (protein, metabolite, or gene with SNT), which is shared between two or more distinct pathways, so that element is specifically attributed to certain pathway and makes a loop on itself. The more elements (proteins, metabolites, genes with SNP) are congregated and focused in certain pathways (node), the larger size of centrality can be expected. The number of nodes with the centrality more than 10 (i.e., the number of edges attached to the node) was equal to 30, while the centrality more than 20 refers to 5 hubs. The centrality reflects the level of occurrence of a biological process. In turn, the occurrence has been was estimated through the frequency of identification of the corresponding molecular factors in the studied samples. The larger centrality indicates the higher frequency of the molecular factor identification.

Studies showed that GRN deficiency is accompanied by increased expression of IL-6, IL-8 and C4 complement factor^33,49^. While IL-8 reduces the production of estrogens^50^, we found estrone among significantly suppressed hormones (Tables 2, 3) involved as in steroidogenesis, as in regulation of dopamine secretion and immune response (Figs. 3, 4). At the same time, C4A, C4B, and C3 are expectedly reduced (Supplementary Appendix C), but the abundance of C4BPA is significantly higher, which may explain the alteration of C4 level. Recent study demonstrated an increased expression of C4 SNP variants and associated escalation of activity of complement system during schizophrenia, which has also been correlated with the reduced synaptic density in post-mortem brains of schizophrenia patients^51^. Immune system bears a huge number of sex hormones receptors, hence, deficient of estrogens (Table 3) reduces the immune response^52^.

We can assume that the leading role of GRN is limited to delaying the activity of microglia for the implementation of neuroimmune signal transmission if neurons are damaged by oxidative stress. Homozygous GNR murine exposed to MPTP exhibited higher density of damaged neurons^53^. Besides, the activity of complement system (C3 and C4 variants) contributes to brain endothelial activation and is related to the perivascular inflammation and associated angiogenesis^54^. Damages, caused by the complement exaggeration, require involvement of microglia to rescue the integrity of the impaired blood–brain barrier and brain microvascular system. Transcriptomic screening of neurons and glia in cerebral cortex displayed the decreased expression of angiogenesis-related genes (*ANGPT2*, FC =  − 1.24, *p* = 5.80e−5; *ANGPTL4*, FC =  − 1.25, *p* = 6.39e−6; *SPARC*, FC =  − 1.77, *p* = 7.55e−9) in patients with schizophrenia^55,56^. Remarkably, but the most recent GWAS study identified rs592927 loci associated with *ANGPTL2* (Supplementary Appendix E) and linked it with the paracrine induction of angiogenesis and acute inflammation reaction^57^.Therefore this loci was ushered as a promising marker for recognition of the early schizophrenia^58^.

In a sense, the current scenario intends to violation of lipids metabolism and manifesting of oxidative stress and immune reactivity (Fig. 3). The deficiency of GRN causes the growing secretion of complement factors by microglia and, thus, tightly associates hsa04080 pathway (neuroactive ligand-receptor binding) through all omics-layers (Fig. 4) by collecting PLMN protein, two SNPs (rs2299542 and rs118125840 for *GRM8* and *GABBR2* glutamate receptors) and metabolites (norepinephrine, dopamine (Supplementary Appendix E), and serotonin (Table 2)). The activity of GRN largely determines the transport to lysosomes, and can designate the increased level of PLMN and GPX3 due to overproduction of ROS. Despite transcriptomic data evidence about the diminished immune response events^59^, the suppression of microglia by GRN, activation of complement system, and overexpression of GPX3 severely argue for the inflammation in schizophrenic patients.

### Is transportation more important than transformation?

Yet, what is the source of pro-oxidants? Our attention focused on the level of FASN as a sign of fatty acid accumulation and the ongoing reduction of triglycerides output, despite previous review noticed the reduced level of polyunsaturated fatty acids but elevated level of lipids peroxidation products^60^ The source of ROS is emerging from the activity of FASN that requires NADPH and, consequently, increases rate of PPP (Fig. 3). Noteworthy, the highest expression of FASN is observed in brain and liver tissues.

Obviously, the increased activity of FASN can be caused by the impaired fatty acids oxidation and glycolysis. In part, that is confirmed by changing in APOB, which indicates the reduced level of VLDL/LDL and the violation of their transport. But here we faced contradictory uncertainty: SORT is a receptor for both GRN and APOB and participates in the transfer of LDL and its further endocytosis. In conditions of the depleted fatty acids transport, one can expect a decrease of activity and surface density of SORT. Oddly, it has been indicated an increase of SORT in patients with schizophrenia^61^. Turning to the panoply of evidence-based data and results obtained in our study, we concluded that the root of pathology is underlined in lipids transport rather than in lipids metabolism.

Occasionally, we found SNP (rs11254275) in *CUBN* loci quite frequently (> 0.88) among studied patients, which permits to locate it together with APOB protein and 7-dehydrocholesterol (vitamin D3) into hsa04977 pathway (Table 3). Due to CUBN is an endocytic receptor, non-synonymous exon polymorphism in *CUBN* gene might be a reason of alternating lipids and apolipoproteins transport and absorption, thus an increased surface density of SORT is considered as a compensatory mechanism. Furthermore, clinical trials and associated genome studies displayed that some splice-variants of *CUBN* cause a deficient of vitamin D transport^62^, whereas C-terminal mutations may induce metabolic syndrome accompanied with severe inflammation^63,64^. Indeed, the resulting trial has many common features with prediabetic conditions because impaired glycolysis and lipids transport are commonly observed features of patients with diabetes mellitus. Moreover, there are reports showing high risk of IR (insulin resistance) and development of T2DM before long following the first episode of schizophrenia had been registered^65,66^, albeit we did not recognize difference in proteome and metabolome between resistant patients and those who suffered the first episode of schizophrenia (Supplementary Appendix A). Does it turn out due to above-mentioned reasons that schizophrenia might be a lysosomal disease associated with a malfunction of lipids transport?

### Cooperation with steroidogenesis

The molecular harmonization between proteome and metabolome merits attention due to several meaningfully observed compounds (Table 2), which are subdivided into lipids (glycocholic acid (GA), androsterone sulfate (AS), cholesterol sulfate (CS), dehydroepiandrosterone (DHEA)), carbohydrates and their derivatives (sphingosine (C16S), 3-*O*-sulfogalactosylceramide (3OSG)) and amines (serotonin (SR), thyroxine (T4), normetanephrine (NMN)). A portion of metabolites (CS, AS, GA, DHEA) is epitomized as a sequential transformation of cholesterol (Table 2, Fig. 3) and associated with loci polymorphisms (Supplementary Appendix E) via hsa04975, hsa04979, hsa04976 pathways.

Cooperation of apolipoproteins with FASN, GPX3, and PEDF in steroidogenesis and in the transport of retinoic acid sharply affects the regulation of the HPA axis. By this reason, the alteration of progesterone and pregnenolone (Table 2) assumingly operates as an indicator of psychopathological changes due to their progenitor activity for neurosteroids that stimulate inflammatory and immune responses. Due to AS (androsterone) conjugate is a substrate for DHEA, it shapes a comprehensive relationship between enzymes of the opposite effect (lyase and transferase), and the ability to convert AS into DHEA and further into estrogens (Fig. 3). Deficiency of aromatase impedes the catalytic conversion of androstenedione and testosterone to estradiol, and NADH is compulsory for this reaction and together with NADPH and oxygen is essential in fatty acids oxidation and PPC (Fig. 3). In this respect, we assume that the detected changes of the circulating DHEA and androsterone sulfate conjugate (Table 3) exhibit symptoms severity, persistence to treatment, and cognitive dysfunction in schizophrenia[NO_PRINTED_FORM].

There is evidence that DHEA exhibits dual pro-oxidative and defending properties achieved through the interaction with PPAR receptors^67^, which are tightly engaged with hsa04024 pathway via cAMP uptake. Admittedly, an increased level of DHEA and consequent stimulation of PPAR/RXR receptors^68^ leads to increased generation of hydroperoxides. The growing risk of oxidative stress is aggravated by FASN and adiponectin upregulation^69^, and following triggering of glutathione, GST, G6PDH, and GPX3 all involved in the metabolism of arachidonic acid (hsa00590) and prostaglandins. At the same time, DHEA is a progenitor for androgens, among which AS and testosterone can be transformed into APOD-stimulating regulation of estrogens, which are antagonized by prolactin (Fig. 3).

Evidence suggests the relationship between DHEA, increased GPX3, and decreased proportion of adipose tissue. A larger volume of adipose tissue positively correlates with FASN activity which fosters the DHEA stimulation^70^. Thus, the accumulation of fatty acids and increased triglycerides, examined in patients with schizophrenia, entails to stimulation of DHEA, AS, their mutual interconversion into estrogens, and subsequent events of lipids uptake via PPAR-α/γ1/γ2 receptors (Fig. 3, Table 2).

Adipocytes represent the main storage of triglycerides as the most oxidized elements. These elements enhance NO generation needed for endothelial permeability and provide protection through the induction of PTGS1. According to GWAS data^71^, the most significant polymorphisms (rs8046 and rs10306166) were associated with hsa00590 pathway and discovered for cyclooxygenase gene PTGS1 (Table 3, Supplementary Appendix E), necessary for PTGH production as an antagonist of thromboxane due to adenylate cyclase stimulating properties. This, possibly, associate both SNPs with the platelet activation (hsa04611) and lipolysis processes (hsa04923), causing elevated norepinephrine generation (Table 2) in patients with schizophrenia^72,73^. Malfunctions of PTGH impact the proper action of PTGS1 and fraught with the angiopathy, and the risk of thrombosis largely frequent among patients with schizophrenia. Hence, the selected elements of hsa0059, hsa04611, and hsa04923 pathways and direct relation of PTGS1 to serotonergic synapse plasticity (Table 3) might be considered as ancient factors of schizophrenia etiology.

### Harmonization with thyroid hormones

Whether DHEA and AS are further boosted along the path of estradiol synthesis? Indeed, if we assume based on the obtained proteomic data that patients with schizophrenia have a complex sign of oxidative stress, then probably smaller fraction of DHEA and AS intend to aromatization (Fig. 3). Hence, aromatase activity and action on adipose tissue would be reduced, whereas prolactin activity, on the contrary, would be increased, which means stimulation of APOD and amplifies the ROS production. Above and beyond, the increased AS (Table 2) requires *SCARB1* as a receptor. Both elements (AS and *SCARB1*) are members of hsa04976 and hsa04979 pathways, however the rs61941687 polymorphism (Table 3, Supplementary Appendix E) negatively affects the activity of *SCARB1* and, this dire consequence is compensated by the increasing density of SORT^74^.

There is no consensus regarding the role of DHEA and AS in the pathogenesis of schizophrenia. Data are mostly contradictory, depending on either the daily cycle or treatment strategy, or other unpredictable effects^75^. There is a trend toward a positive association between DHEA and the severity of schizophrenia, which mostly fits the interface highlighted in this study. The PPAR-mediated metabolism of lipids (Figs. 3, 4) is highly dependent on selenium and requires cooperative action of DHEA and thyroid hormones. However, the overproduction of T4 and T3 does not affect the DHEA-dependent PPAR-signaling^76^. Thereof, T4 deficiency and DHEA overproduction are common symptoms attended by oxidative stress and pro-inflammatory condition at schizophrenia^77^.

Due to the synthesis of T4 and functioning of GPX3 are determined by selenium, we cannot bypass the potential role of SELENOP, which interconnects GPX3 activity with thyroid hormones releasing^78^ (Fig. 3, Table 1). Its responsibilities are focused on the transfer of selenium in the tissues, including the brain, and antioxidant functions. The observed elevation (Supplementary Appendix C) can be caused by a higher demand of selenium due to both upregulated GPX3 and T4 biosynthesis on the background of subclinical hypothyroidism.

There have been far few studied about the positive association between thyroid-stimulating hormone (TSH) and prolactin but symptoms of hyperprolactinemia on the background of hypothyroidism are usually observed^79^. In this study, we found cautioning reduction of L-3,5-diiodotyrosine and thyroxine hormones (Table 2) interconnected with dopamine and catecholamines uptake (hsa00350 and hsa04918 pathways; Fig. 4). But only GPX3 with inclusion of two SNPs (rs8046 and rs10306166) is co-associated with the regulation of thyroid hormones synthesis and considered as an entering point of arachidonic acid metabolism and serotonergic synapse transmission (Table 3). Other studies indicated a relationship between the severity of schizophrenia and the diminished synthesis of T3 andT4, and T4 is lowering more apparent. However, there was no difference in TSH compared to the control group^80^.

The tacit assumption is that subclinical hypothyroidism is regular for schizophrenia patients, but some research witnesses about its association with antipsychotic medication^81^. There is no unambiguous opinion, but T4 increases gradually at schizophrenia, although data were related to the first hospitalization and attributed to stressful situation as a consequence of neuroprotective modulation^81^. Generally, there is a gentle tendency to the decrease of T4, which positively affects the prolactin level in patients with schizophrenia^80,81^. In turn, prolactin sends a positive effect on APOD and inhibits adiponectin, thus reducing the concentration of aromatic steroids. Due to estradiol inversely correlates with oxidative stress (through FASN and APOD; Fig. 3), it greatly contributes to the increased risk of oxidative stress.

Insofar our concept fits into the deployed proteomic-metabolic framework (Figs. 3, 4), we encouraged further investigation of association between serotonin, tyrosine, T4 and dopamine. It is known that the elevated dopamine entails to the decrease of TSH and T4. This is supported by the data for therapy by the dopamine receptor blockers leading to subclinical hypothyroidism and hyperprolactinemia that causes the elevation of TRH.

There are clinical observations establish a strong negative correlation between 5-hydroxyindolylacetic acid (5-HIAA) with T3 and TSH in patients with schizophrenia. It is essential to understand that T4 and TSH impacts nor serotonin secretion per se but the population (density) of serotonin receptors and, therefore, their vulnerability to serotonin^82,83^. Substitutional therapy of schizophrenia with TSH and treatment of depression with T4 reliably improves amelioration of patients and delivers a positive effect on the level of serotonin due to the increased perceptivity of 5-HT receptors^84^. Otherwise, the increase of 5-HIAA is recognized due to a stoichiometric shift between serotonin and its receptors towards the ligand of the receptor.

Normetanephrine as a catecholamine (Supplementary Appendix D), is a deactivated product of norepinephrine, and a product of tyrosine transformation. There are extensive data on the substantial role of catecholamines in the severity of schizophrenia^85,86^. The axis of norepinephrine and serotonin via diencephalon into the limbic system and the cerebral cortex plays a considerable role emotional component of human’s life. Patients with the compromised noradrenergic transmission are characterized by the increased T3 in brain nuclei since norepinephrine is a T3 co-transmitter in noradrenergic signal transduction^87^.

Thus, depletion of catecholamines typically accompanies a deficiency of serotonin. Indeed, the level of norepinephrine and normetanephrine is lower in patients with schizophrenia (Supplementary Appendix D). There is considerable evidence that the decrease is caused by the reduced activity of brain monoamine oxidase (MAO-B) in patients with schizophrenia^88^, which is complementary with our data. In this respect, the role of estrogen in schizophrenia pathogenesis is severely important, since estrogen positively regulates dopamine activity. Thus the deficiency in estrogen obviously entails to the increased catechol-*O*-methyltransferase activity and, consequently, to the decreased dopaminergic transmission. That is the reason of why, medication in combination with estrogens restores dopamine receptors sensibilization and provides amelioration of psychotic symptoms^89^.

We admit, that medications used for schizophrenia treatment may occasionally produce a significant impact on the reconstructed image of the molecular event in the proposed connective model. Still, the complete view is doubtful due to the high complexity of schizophrenia pathology; however, the interplay between the functions of lipids transport/metabolism, thyroid hormone synthesis, and steroidogenesis more than merely evident (Fig. 4).

## Conclusion

Up to nowadays, diagnostics of schizophrenia is burden of responsibility of the expertized physician. The lack of clinically relevant serological or metabolic markers is fraught with difficulties in differentiation of schizophrenia from bipolar disorder or depression syndrome. In this study, we tried to combine three interplaying layers (genome, proteome, and metabolome) to overview molecular events at schizophrenia. Although the majority of data were extracted from the metabolic and proteomic assay, because the GWAS data are beyond the scope of the confidence, yet they sufficiently play a symphony of events, which are involved in the etiology and pathogenesis of schizophrenia. The proposed graph (Fig. 4) consolidates extracted biological pathways that cover defined metabolites and proteins, and integrates elements omitted in the study but previously associated with schizophrenia. Data collected in the study reflect a multifaced chain of proportional events that instruct the immune response, compromised lipids transport, and signs of lysosomal accumulation. The largest centralities (Fig. 4) integrate proteome re-arrangements that are accompanied by manifested impairment os steroidogenesis and hypothyroidism, which are tightly linked with cAMP-mediated signaling and tyrosine metabolism. These processes affect the perceptivity of 5-HT receptors and thyroid-supplemented noradrenergic transmission.

It seems that not so much lipids metabolism (including PPAR signaling) but lipids transport contributes in disturbance of biological processes in schizophrenic patients, which supports the relation of schizophrenia to neurodegenerative disease. On the other hand, imbalance in steroidogenesis (Table 2) and related proteins (Table 1) favors over imbalance in thyroid-related and catecholamines endocrine axis. It is hard to overestimate the role of steroids, thyroid hormones, and catecholamines, because all of them are covered by neuroactive ligand-receptor interaction (one of five largest centrality; Fig. 4) and at the same they spread among a few more specialized centralities including estrogen signaling, axon guidance, glutamatergic and dopaminergic synapse plasticity (Fig. 4).

A series of independent investigations elsewhere on significantly larger cohort of patients with schizophrenia is essential and can provide a vanquish above the obvious limitations of this study. No words exaggerating the performance of such collaboration to consolidate proteomic, metabolomic, and genotyping for rigorous reconstruction of probably one of the most enigmatic diseases. It is even much more feasible if render a convolutional neural network for proteomic and metabolomic data that recently has been successfully realized for the classification and determination of schizophrenia comorbidity^90^ as a tool complementary to the conventional systematic approach.

## Limitations

Apart from the small size of study population even together with the validating cohort, the background derived from multidrug therapy is by far one of the main challenging tasks in research of schizophrenia. Treatment strategy may cause a great influence on the final result and may require correction actions to align between patient’s assessment and metabolic background that shadowed by medications. In this study we draw the attention on the huge number of drug and drug-like metabolites observed in serum samples which were excluded from the consideration and required additional statistical actions to avoid biases.

## Materials and methods

### Reagents

Urea (99%) and formic acid (98%+, pure) were obtained from Acros Organics (Geel, Belgium). Trifluoroacetic acid (99%, Reagent Plus®), triethylammonium bicarbonate (1 M solution), 4-vinylpyridine (95%), sodium deoxycholic acid (> 97% titration) were from Sigma (St. Louis, MO, USA). Acetonitrile (HPLC grade, filtered for 0.2 µm) was purchased from Fisher Chemical (Loughborough, UK). Acetic acid (EMSURE®, glacial, anhydrous for analysis) was from Merck (Darmstadt, Germany). TCEP (Tris(2-carboxyethyl) phosphine hydrochloride) was purchased from Pierce™ (Thermo Fisher, Rockford, IL, USA). Trypsin (sequencing grade modified), 5 vials × 20 µg, lyophilized powder (Promega; Madison, WI, USA). β‐glucuronidase from *Escherichia coli* K12 strain, 5 ml falcon vial, specific activity ~ 140 U/mg at 37 °C (Roche Diagnostics GmbH; Germany). Water (TOC < 3 ppb) was obtained from Milli-Q Integral 3 purification system, Millipore S.A.S (France). Oasis® 3 cc (cubic centimeter) nominal volume, 60 mg resin, type of resin: WAX—weak anion exchange (Waters Corporation; Milford, MA, USA). d5-betamethasone, 1 mg, powder (catalogue number: B327002; Toronto Research Chemicals; Toronto, ON, Canada). UPS-2—Universal Proteomics Dynamic Range Standard, 10.6 µg, dynamic range: 0.5 fmoles—50,000 fmoles; 48 different proteins (Sigma; Saint Louis, MO, USA).

### Ethical consideration

The study design was approved by the local Ethical Committee of the Alexeev N.A. 1st Clinics of Mental Health, (Moscow; AXM-EH2019-R017.G12 issued on February 15, 2019; AXM-EH2020-R004.Y04 issued on March 4, 2020). All handlings and use of material were provided according to the WMA Declaration of Helsinki on Ethical Principles for Medical Research Involving Human Subjects (revision Fortaleza, 2013). All the participants were aware of the research purpose. Informed consent was obtained from all participants of the control group and informed consent was obtained from a parent and/or legal guardian of participants of the assay group.

### Subjects

We enrolled totally n = 127 subjects for this study among which n = 49 subjects were being schizophrenic subjects who were accepted for inpatient, n = 50 were healthy volunteers aligned by anthropometric data (age, genders ration, BMI). Additionally, n = 28 schizophrenic subjects and n = 11 healthy volunteers (n = 39 subjects in total) were recruited for rendering of the validation study (Table 4). Subjects matched for age and gender ratio and were examined in terms of severity of catatonic, positive and general symptoms, and degree of cognitive impairments. Most of the observed patients with schizophrenia (~ 60%) burden a prevalence of hereditary loading. Details of clinical and psychometric characteristics are in Supplementary Appendix A.

**Table 4:** Sociodemographic, clinical and psychometric characteristics of patients with schizophrenia and group of healthy volunteers participated in the study.

Parameter or feature		Patients with schizophrenia		Control group	
		Study cohort	Validating cohort	Study cohort	Validating cohort
Size (n)		49	28	50	11
Gender, n (%)	Males	26 (53)	15 (53)	19 (38)	5 (45)
	Females	23 (47)	13 (47)	31 (62)	6 (55)
Age, mean + SD	Current age	26.9 ± 5.2	28.4 ± 7.2	25.9 ± 5.8	26.2 ± 4.1
	At onset of prodromal symptom	17.6 ± 7.4	17.8 ± 7.1	N/A	N/A
	At manifested syndrome	22.8 ± 7.0	20.5 ± 7.4		
	At onset of first psychotic symptoms	21.9 ± 8.6	20.6 ± 7.3		
	At first hospitalization	22.3 ± 8.6	22.3 ± 6.8		
Duration, years	Duration from prodrome	9.3 ± 6.9	10.6 ± 6.9	N/A	N/A
	Duration from manifestation	4.1 ± 5.3	7.9 ± 6.9		
Education level, n (%)	Incomplete secondary school	2 (4)	3 (11)	1 (2)	0 (0)
	Secondary school	8 (16)	7 (25)	15 (30)	2 (19)
	Vocational school	13 (27)	11 (39)	1 (2)	1 (9)
	Incomplete high school	7 (14)	2 (7)	4 (8)	1 (9)
	High school	19 (39)	5 (18)	29 (58)	7 (63)
Marital status, n (%)	Married	6 (12)	1 (3.5)	15 (30)	5 (45)
	Single	3 (6)	26 (93)	3 (6)	2 (19)
	Divorced	40 (82)	1 (3.5)	32 (64)	4 (36)
Occupation, n (%)	Student	10 (20)	6 (21)	37 (74)	5 (45)
	Employed	13 (27)	5 (18)	13 (26)	6 (55)
	Unemployed	18 (37)	17 (61)	0 (0)	0 (0)
	Disabled	8 (16)	0 (0)	0 (0)	0 (0)
Hereditary loading, n (%)	Yes	30 (61)	13 (46)	N/A	N/A
	No	19 (39)	15 (54)		
PANSS score	Total	112.5	102.7	33.1	30.7
	Positive	29.8	25.2	8.9	7.3
	Negative	28.9	31.1	7.4	6.9
	General	53.8	46.4	16.8	16.2
BFCR scale		10.5	7.9	0	0
NSA-4		21.8	20.9	0	0
SAS		1.2	0.74	0	0
DSM-5 score		14.8	14.8	0	0
FAB score		15.7	13.6	18	18

*PANSS* positive and negative syndrome scale, *BFCR scale* Bush-Francis Catatonia Rating Scale, *NCS4* the 4-item negative symptom assessment, *SAS* Simpson-Angus Scale, *DSM-5* Diagnostic and Statistical Manual of mental disorders, fifth edition, *FAB* Frontal Assessment Battery.

*p < 0.05 Mann–Whitney test.

**t-test, statistically significant.

We selected patients in both study and validating cohorts (Table 1) aligned in the majority of sociodemographic and psychometric parameters. These two groups matched in age at onset of prodromal symptom, at manifested syndrome, at onset of first psychotic symptoms and at first hospitalization where statistical significance exceeded p > 0.83 meaning the lack of differences between patients. The same matches were reached in regard of psychometric characteristics based on the main scales and scores routinely used in clinical psychiatry (PANSS, BFCR, NCS4 and DSM-5). The attained alignment legalized employment of the validating group to control and correct, if necessary, the hypothesized molecular mechanisms originally extracted from the proteome, metabolome and genome-wide associated studies of the assayed group of patients with schizophrenia disputed it was combined from those who had no previous testimony of the disease history and those who had been qualified as resistant to medication and therapy for a long time (more than 10 years) from the manifestation.

The control group consisted of healthy volunteers, matched by age and gender, who had no history of mental disorders and were not on any medications; none of the respondents had a history of serious physical illness, including cardiac, cancer or neurological disorders.

### Sample handling

Proteomic analysis of plasma samples was performed as described in Ref.^91^. Briefly, we used 100 µg of protein fraction (about 2 µL) for proceeding and digestion with trypsin. Details of proteomic samples preparation are available in Supplementary Appendix-B (section no. 2). Metabolomic analysis was accomplished as described in Ref.^92^ with minor modifications. Briefly, 100 µL of plasma was fortified with *d5*-betamethasone (ISTD) and supplied with SPE/LLE extraction. Details of metabolomic sample preparation are available in Supplementary Appendix B (section no. 4).

### Proteomic and metabolomic study

Proteomic analysis was performed on an ultra-high-resolution Orbitrap Fusion mass spectrometer equipped with a nanoflow Ultimate 3000 UPLC (both from Thermo Fisher, Germany) as described in Ref.^91^. A survey of metabolomic compounds was conducted on a high-resolution G6550 Q-TOF mass spectrometer with a 1290 Infinity UPLC (both from Agilent, Germany). Details are given in Supplementary Appendix B (sections no. 3 and no. 7 for proteomic analysis, and sections no. 5 and no. 8 for metabolomic analysis). The mass spectrometry proteomics data have been deposited to the ProteomeXchange Consortium via the PRIDE^93^ partner repository with the dataset identifier PXD035863 and 10.6019/PXD035863.

### Genome-wide association study

Case (48 patients with schizophrenia) and control “1000 Genomes Phase 3” (200 volunteers) are taken from the same European population. Total DNA was isolated using QIAamp DNA (Qiagen, Germany). Case (48 patients with schizophrenia) and control “1000 Genomes Phase 3” (200 volunteers) are taken from the same European population. Sample handled on an Illumina HiScan (San Diego, CA, USA) and genotyped for 652,297 markers for 231 subjects, of which 48 cases and 183 controls, on an Infinium Global Chips Screening Array-24v2.0 using the IMPUTE2 program with parameters Ne = 20,000 and k = 90. Additional filtering was performed by metrics INFO > 0.5 and genotype probability > 0.5. Quality Control allowing only 2% of missing SNPs was performed separately in each individual dataset and Association Search was conducted using the plink version 1.90b6.7 program. The GWAS of patients with schizophrenia was performed using logistic regression with imputation probabilities adjusted for PCA covariates, chosen as nominally significant with p < 0.05 in the employed logistic regression. Control of the homogeneity of the obtained samples was carried out using a PCA made by smart-PCA version13050 EIGENSOFT version 7.2.0 package^94^ (Supplementary Appendix E). To avoid overloading the GWAS by adding too many covariates to the regression model, only the first 10 principal components were considered and tested for inclusion. The final set of covariates included the first five principal components as recommended for most GWAS approaches. Details of the analysis and results are given in Supplementary Appendix B (section no. 9) and Appendix E.

### Quantitative analysis

Quantitative analysis for proteomics was performed as described in Ref.^95^. The UPS-2 (Sigma; Saint Louis, MO, USA) employed to plot the calibration curve and fit the meaningful proteins. Details of quantitation are acknowledged in Supplementary Appendix B (section no. 6).

#### Statistics

Statistical analysis was performed on an R-package, and complete details are available in Supplementary Appendix B (section no. 10). The significance of anthropometric and psychometric items between the studied groups was performed by a two-sided *t*-test at a *p*-value cut-off *p* < 0.05. Due to the small size of the studied groups, significance in the measured concentrations of proteins and metabolites was evaluated by Wilcoxon’s test with significance level cut-off p < 0.05. To achieve data reduction and discrimination, principal component analysis (PCA) and pairwise Student’s *t*-test at *p* < 0.05 were applied to the total proteome and metabolome identified in both control and assayed (subjects with schizophrenia) groups. To perform variable selection and classification of the studied cohort, sparce partial least-squares discriminant analysis (sPLS-DA) with 0.95 ellipse area confidence level was utilized. To be selected from the total proteome, the candidate protein shall meet the criterion of unicity when more than one (ni(p) > 1) unique peptide shall be met for the certain protein among the totality of peptides Acceptance criteria for the protein identification were based on the Human Proteome Project Mass Spectrometry Data Interpretation Guidelines 3.0^96^. NSAFs (Normalized Spectra Abundance Factor) were summarized for each protein within the studied groups, and data with zero means were imputed. A measure of protein abundance was represented as a median value fold changes (FC) ratio toward the control group and calculated based on the absolute concentration sampled from the UPS-2 (see Supplementary Appendix B for details). For metabolites, the criteria of frequency exceeding one occasion within each studied group were significant. The significance scores obtained after identification of metabolite compounds (Supplementary Appendix B section no. 8 for more details) were averaged within each studied group, and zero means were imputed. Proteins and metabolites with a frequency exceeding 0.85 and the fold changes cut-off > 2 or < 0.5 at a p-value less than 0.05 were considered as significant in quantitative property. To revealed outliers and significant differences in a quantitative loading we applied Wilcox test with Benjamini–Hochberg correction for multiple hypothesis testing, and the adjusted *p*-values were plotted on Volcano against the calculated fold changes. Proteins and metabolites, significantly altered between the studied groups, were extracted and submitted for functional and pathways annotation analysis at a q-value threshold less than 0.01 using Gene Ontology (GO) toolset against the complete human genome as a reference list. The enriched terms were refined with a similarity coefficient of > 0.7 to remove the redundant terms. The refined terms were associated with the protein and metabolite lists drawn up for the studied groups and adjusted with KEGG means and Reactome pathway terms to consolidate observed proteins, metabolites and associated loci with the current knowledge into centrality graph. All other analyses were performed with the in-house scripts written in R (version 3.2.0; R Foundation for Statistical Computing, Vienna, Austria; https://www.r-project.org/).

## Supplementary Information

Supplementary Information 1.

Supplementary Information 2.

Supplementary Information 3.

Supplementary Information 4.

Supplementary Information 5.

## Data Availability

The datasets generated and/or analyzed during the current study are available in the PRIDE^93^ (Proteome Exchange) repository under registered ID: PXD035863 (or following link: 10.6019/PXD035863).

## References

[1] Tandon R (2013). Definition and description of schizophrenia in the DSM-5. Schizophr. Res..

[2] Miyamoto S, Miyake N, Jarskog LF, Fleischhacker WW, Lieberman JA (2012). Pharmacological treatment of schizophrenia: A critical review of the pharmacology and clinical effects of current and future therapeutic agents. Mol. Psychiatry.

[3] Oh H, Koyanagi A, Kelleher I, DeVylder J (2018). Psychotic experiences and disability: Findings from the Collaborative Psychiatric Epidemiology Surveys. Schizophr. Res..

[4] Mattila T (2015). Impact of DSM-5 changes on the diagnosis and acute treatment of schizophrenia. Schizophr. Bull..

[5] Biedermann F, Fleischhacker WW (2016). Psychotic disorders in DSM-5 and ICD-11. CNS Spectr..

[6] Shorter KR, Miller BH (2015). Epigenetic mechanisms in schizophrenia. Prog. Biophys. Mol. Biol..

[7] Dudley E, Hässler F, Thome J (2011). Profiling for novel proteomics biomarkers in neurodevelopmental disorders. Expert Rev. Proteom..

[8] Iavarone F (2014). Characterization of salivary proteins of schizophrenic and bipolar disorder patients by top-down proteomics. J. Proteom..

[9] Schwarz E (2012). Identification of a biological signature for schizophrenia in serum. Mol. Psychiatry.

[10] Rodrigues-Amorim D (2019). Proteomics in schizophrenia: A gateway to discover potential biomarkers of psychoneuroimmune pathways. Front. Psychiatry.

[11] Borgmann-Winter KE (2020). The proteome and its dynamics: A missing piece for integrative multi-omics in schizophrenia. Schizophr. Res..

[12] Martins-de-Souza D (2014). Proteomics, metabolomics, and protein interactomics in the characterization of the molecular features of major depressive disorder. Dialogues Clin. Neurosci..

[13] Chiam JTW, Dobson RJB, Kiddle SJ, Sattlecker M (2015). Are blood-based protein biomarkers for Alzheimer’s disease also involved in other brain disorders? A systematic review. J. Alzheimers Dis..

[14] Arnold SE, Ruscheinsky DD, Han LY (1997). Further evidence of abnormal cytoarchitecture of the entorhinal cortex in schizophrenia using spatial point pattern analyses. Biol. Psychiatry.

[15] Magdalon J (2020). Complement system in brain architecture and neurodevelopmental disorders. Front. Neurosci..

[16] Mayilyan KR, Weinberger DR, Sim RB (2008). The complement system in schizophrenia. Drug News Perspect..

[17] Choudhari S, Patange A, Mara B, Mutalik N, Chaudhari R (2017). Fractional protein study in chronic schizophrenics—A case control study 1. Med. Innov..

[18] Severance EG, Yolken RH (2016). Role of immune and autoimmune dysfunction in schizophrenia. Handb. Behav. Neurosci..

[19] Nucifora LG (2019). Increased protein insolubility in brains from a subset of patients with schizophrenia. Am. J. Psychiatry.

[20] Owens SJ, Murphy CE, Purves-Tyson TD, Weickert TW, Shannon Weickert C (2018). Considering the role of adolescent sex steroids in schizophrenia. J. Neuroendocrinol..

[21] Cesková E, Prikryl R, Kaspárek T (2007). Testosterone in first-episode schizophrenia. Neuro Endocrinol. Lett..

[22] Ramanathan S, Miewald J, Montrose D, Keshavan MS (2015). Can age at sexual maturity act as a predictive biomarker for prodromal negative symptoms?. Schizophr. Res..

[23] Boiko AS (2020). Cortisol and DHEAS related to metabolic syndrome in patients with schizophrenia. Neuropsychiatr. Dis. Treat..

[24] Brand BA, de Boer JN, Sommer IEC (2021). Estrogens in schizophrenia: Progress, current challenges and opportunities. Curr. Opin. Psychiatry.

[25] Bergemann N, Parzer P, Runnebaum B, Resch F, Mundt C (2007). Estrogen, menstrual cycle phases, and psychopathology in women suffering from schizophrenia. Psychol. Med..

[26] Tkachev D, Mimmack ML, Huffaker SJ, Ryan M, Bahn S (2007). Further evidence for altered myelin biosynthesis and glutamatergic dysfunction in schizophrenia. Int. J. Neuropsychopharmacol..

[27] Comes AL (2018). Proteomics for blood biomarker exploration of severe mental illness: Pitfalls of the past and potential for the future. Transl. Psychiatry.

[28] Li Y (2012). Label-free quantitative proteomic analysis reveals dysfunction of complement pathway in peripheral blood of schizophrenia patients: Evidence for the immune hypothesis of schizophrenia. Mol. Biosyst..

[29] Nascimento JM, Martins-de-Souza D (2015). The proteome of schizophrenia. NPJ Schizophr..

[30] Timsina J (2022). Comparative analysis of Alzheimer’s disease cerebrospinal fluid biomarkers measurement by multiplex SOMAscan platform and immunoassay-based approach. J. Alzheimers Dis..

[31] Korolainen MA, Nyman TA, Aittokallio T, Pirttilä T (2010). An update on clinical proteomics in Alzheimer’s research. J. Neurochem..

[32] Papapetropoulos A, García-Cardeña G, Madri JA, Sessa WC (1997). Nitric oxide production contributes to the angiogenic properties of vascular endothelial growth factor in human endothelial cells. J. Clin. Investig..

[33] Perkins DO (2015). Towards a psychosis risk blood diagnostic for persons experiencing high-risk symptoms: Preliminary results from the NAPLS project. Schizophr. Bull..

[34] Russell JK, Jones CK, Newhouse PA (2019). The role of estrogen in brain and cognitive aging. Neurotherapeutics.

[35] Sun J, Walker AJ, Dean B, van den Buuse M, Gogos A (2016). Progesterone: The neglected hormone in schizophrenia? A focus on progesterone-dopamine interactions. Psychoneuroendocrinology.

[36] Schwarz E (2010). Validation of a blood-based laboratory test to aid in the confirmation of a diagnosis of schizophrenia. Biomark. Insights.

[37] Gibbons AS (2011). The neurobiology of APOE in schizophrenia and mood disorders. Front. Biosci. (Landmark Ed.).

[38] González-Castro TB (2015). No association between ApoE and schizophrenia: Evidence of systematic review and updated meta-analysis. Schizophr. Res..

[39] Xuan J (2011). Metabolomic profiling to identify potential serum biomarkers for schizophrenia and risperidone action. J. Proteome Res..

[40] Lan MJ (2009). Metabonomic analysis identifies molecular changes associated with the pathophysiology and drug treatment of bipolar disorder. Mol. Psychiatry.

[41] Martins-de-Souza D (2009). Prefrontal cortex shotgun proteome analysis reveals altered calcium homeostasis and immune system imbalance in schizophrenia. Eur. Arch. Psychiatry Clin. Neurosci..

[42] Valdés-Tovar M (2022). Insights into myelin dysfunction in schizophrenia and bipolar disorder. World J. Psychiatry.

[43] Martins-de-Souza D (2010). Proteome analysis of the thalamus and cerebrospinal fluid reveals glycolysis dysfunction and potential biomarkers candidates for schizophrenia. J. Psychiatr. Res..

[44] Gass J (2012). Progranulin regulates neuronal outgrowth independent of sortilin. Mol. Neurodegener..

[45] Filiano AJ (2013). Dissociation of frontotemporal dementia-related deficits and neuroinflammation in progranulin haploinsufficient mice. J. Neurosci..

[46] Settembre C (2011). TFEB links autophagy to lysosomal biogenesis. Science.

[47] Tanaka Y, Chambers JK, Matsuwaki T, Yamanouchi K, Nishihara M (2014). Possible involvement of lysosomal dysfunction in pathological changes of the brain in aged progranulin-deficient mice. Acta Neuropathol. Commun..

[48] Martens LH (2012). Progranulin deficiency promotes neuroinflammation and neuron loss following toxin-induced injury. J. Clin. Investig..

[49] Chen J (2015). Comparative proteomic analysis of plasma from bipolar depression and depressive disorder: Identification of proteins associated with immune regulatory. Protein Cell.

[50] Lebrun CEI (2005). Endogenous oestrogens are related to cognition in healthy elderly women. Clin. Endocrinol. (Oxf.).

[51] Sekar A (2016). Schizophrenia risk from complex variation of complement component 4. Nature.

[52] Barrueto L (2020). Resistance to checkpoint inhibition in cancer immunotherapy. Transl. Oncol..

[53] Yin F (2010). Exaggerated inflammation, impaired host defense, and neuropathology in progranulin-deficient mice. J. Exp. Med..

[54] Katsel P, Roussos P, Pletnikov M, Haroutunian V (2017). Microvascular anomaly conditions in psychiatric disease. Schizophrenia—Angiogenesis connection. Neurosci. Biobehav. Rev..

[55] Katsel P, Davis KL, Gorman JM, Haroutunian V (2005). Variations in differential gene expression patterns across multiple brain regions in schizophrenia. Schizophr. Res..

[56] Zhang Y (2014). An RNA-sequencing transcriptome and splicing database of glia, neurons, and vascular cells of the cerebral cortex. J. Neurosci..

[57] Amadatsu T (2016). Macrophage-derived angiopoietin-like protein 2 exacerbates brain damage by accelerating acute inflammation after ischemia-reperfusion. PLoS ONE.

[58] Guo S (2021). Genome wide association study identifies four loci for early onset schizophrenia. Transl. Psychiatry.

[59] Morgan LZ (2016). Quantitative trait locus and brain expression of HLA-DPA1 offers evidence of shared immune alterations in psychiatric disorders. Microarrays.

[60] Davison J, O’Gorman A, Brennan L, Cotter DR (2018). A systematic review of metabolite biomarkers of schizophrenia. Schizophr. Res..

[61] Boggild S, Molgaard S, Glerup S, Nyengaard JR (2016). Spatiotemporal patterns of sortilin and SorCS2 localization during organ development. BMC Cell Biol..

[62] Ciancio JIR, Furman M, Banka S, Grunewald S (2019). Profound vitamin D deficiency in four siblings with Imerslund–Grasbeck syndrome with homozygous CUBN mutation. JIMD Rep..

[63] Fyfe JC (2018). Inherited selective cobalamin malabsorption in Komondor dogs associated with a CUBN splice site variant. BMC Vet. Res..

[64] Bedin M (2020). Human C-terminal CUBN variants associate with chronic proteinuria and normal renal function. J. Clin. Investig..

[65] Guest PC (2010). Increased levels of circulating insulin-related peptides in first-onset, antipsychotic naïve schizophrenia patients. Mol. Psychiatry.

[66] De Hert M (2006). Prevalence of diabetes, metabolic syndrome and metabolic abnormalities in schizophrenia over the course of the illness: A cross-sectional study. Clin. Pract. Epidemiol. Ment. Health.

[67] Fujita A (2002). In vivo activation of the constitutive androstane receptor beta (CARbeta) by treatment with dehydroepiandrosterone (DHEA) or DHEA sulfate (DHEA-S). FEBS Lett..

[68] Goodarzi MO, Carmina E, Azziz R (2015). DHEA, DHEAS and PCOS. J. Steroid Biochem. Mol. Biol..

[69] Karbowska J, Kochan Z (2005). Effect of DHEA on endocrine functions of adipose tissue, the involvement of PPAR gamma. Biochem. Pharmacol..

[70] Mayas MD (2010). Inverse relation between FASN expression in human adipose tissue and the insulin resistance level. Nutr. Metab. (Lond.).

[71] Shungin D (2015). New genetic loci link adipose and insulin biology to body fat distribution. Nature.

[72] van Kammen DP, Kelley M (1991). Dopamine and norepinephrine activity in schizophrenia. An integrative perspective. Schizophr. Res..

[73] Nagamine T (2020). Role of norepinephrine in schizophrenia: An old theory applied to a new case in emergency medicine. Innov. Clin. Neurosci..

[74] Huang C-J (2018). Schizophrenia in type 2 diabetes mellitus: Prevalence and clinical characteristics. Eur. Psychiatry.

[75] Misiak B (2018). Testosterone, DHEA and DHEA-S in patients with schizophrenia: A systematic review and meta-analysis. Psychoneuroendocrinology.

[76] Prough RA, Clark BJ, Klinge CM (2016). Novel mechanisms for DHEA action. J. Mol. Endocrinol..

[77] Mancini A (2016). Thyroid hormones, oxidative stress, and inflammation. Mediat. Inflamm..

[78] Oo SM (2018). Serum selenoprotein P, but not selenium, predicts future hyperglycemia in a general Japanese population. Sci. Rep..

[79] Halbreich U, Kinon BJ, Gilmore JA, Kahn LS (2003). Elevated prolactin levels in patients with schizophrenia: Mechanisms and related adverse effects. Psychoneuroendocrinology.

[80] Marian G, Nica EA, Ionescu BE, Ghinea D (2009). Hyperthyroidism—Cause of depression and psychosis: A case report. J. Med. Life.

[81] Akiibinu MO, Ogundahunsi OA, Ogunyemi EO (2012). Inter-relationship of plasma markers of oxidative stress and thyroid hormones in schizophrenics. BMC Res. Notes.

[82] Tejani-Butt SM, Yang J, Kaviani A (1993). Time course of altered thyroid states on 5-HT1A receptors and 5-HT uptake sites in rat brain: An autoradiographic analysis. Neuroendocrinology.

[83] Bauer M, Heinz A, Whybrow PC (2002). Thyroid hormones, serotonin and mood: Of synergy and significance in the adult brain. Mol. Psychiatry.

[84] Gan Z (2019). Rapid cycling bipolar disorder is associated with antithyroid antibodies, instead of thyroid dysfunction. BMC Psychiatry.

[85] Yamamoto K, Hornykiewicz O (2004). Proposal for a noradrenaline hypothesis of schizophrenia. Prog. Neuropsychopharmacol. Biol. Psychiatry.

[86] Carlsson A (2001). Interactions between monoamines, glutamate, and GABA in schizophrenia: New evidence. Annu. Rev. Pharmacol. Toxicol..

[87] Zhao T, Chen BM, Zhao XM, Shan ZY (2018). Subclinical hypothyroidism and depression: A meta-analysis. Transl. Psychiatry.

[88] Camarena B (2012). Monoamine oxidase a and B gene polymorphisms and negative and positive symptoms in schizophrenia. ISRN Psychiatry.

[89] Dumas JA, Makarewicz JA, Bunn J, Nickerson J, McGee E (2018). Dopamine-dependent cognitive processes after menopause: The relationship between COMT genotype, estradiol, and working memory. Neurobiol. Aging.

[90] Kopylov AT (2021). Convolutional neural network in proteomics and metabolomics for determination of comorbidity between cancer and schizophrenia. J. Biomed. Inform..

[91] Kopylov AT (2020). Revelation of proteomic indicators for colorectal cancer in initial stages of development. Molecules.

[92] Piper T (2020). Detecting the misuse of 7-oxo-DHEA by means of carbon isotope ratio mass spectrometry in doping control analysis. Rapid Commun. Mass Spectrom..

[93] Perez-Riverol Y (2022). The PRIDE database resources in 2022: A hub for mass spectrometry-based proteomics evidences. Nucleic Acids Res..

[94] Patterson N, Price AL, Reich D (2006). Population structure and eigen analysis. PLoS Genet..

[95] Kopylov AT (2020). Association of proteins modulating immune response and insulin clearance during gestation with antenatal complications in patients with gestational or type 2 diabetes mellitus. Cells.

[96] Deutsch EW (2019). Human proteome project mass spectrometry data interpretation guidelines 3.0. J. Proteome Res..

