# Supplementary material for: Consolidation of metabolomic, proteomic, and GWAS data in connective model of schizophrenia
Source: Sci Rep. 2023 Feb 6;13:2139. doi: 10.1038/s41598-023-29117-7 (PMC9901842; doi:10.1038/s41598-023-29117-7)
Supplement: Supplementary file 1 — Supplementary Information 1. [file 41598_2023_29117_MOESM1_ESM.pdf]

Appendix A

Demography: sociodemographic, anthropometric and psychometric commentaries and characteristics of patients with schizophrenia (study cohort and validating cohort) and control group of healthy volunteers.

Authors: Arthur T. Kopylov, Alexander A. Stepanov, Tatiana V. Butkova, Kristina A. Malsagova, Natalia V. Zakharova, Georgy P. Kostyuk, Artem U. Elmuratov, Anna A. Kaysheva

Connective model of schizophrenia: a roadmap in maze of metabolomic, proteomic and GWAS data

Arthur T. Kopylov, Alexander A. Stepanov, Tatiana V. Butkova, Kristina A. Malsagova, Natalia V. Zakharova, Georgy P. Kostyuk, Artem U. Elmuratov, Anna A. Kaysheva

Appendix A: Demography

Content

1. Subjects: pilot elucidation and segregation 1
2. Subjects: combination of groups for the main consideration 3

1. Subjects: pilot elucidation and segregation

We enrolled totally n=127 subjects for this study among which n=49 were being schizophrenic subjects who were accepted for inpatient, n=50 were healthy volunteers aligned by anthropometric data (age, genders ration, BMI etc.) and additionally n=28 schizophrenic subjects which were recruited for rendering of the validation study.

Initially, the study population of primary schizophrenic subjects (n=49) was divided in two subgroups comprised of patients with the first episode of schizophrenia (n=24, Group A) and patients resistant to medication and suffering a long-term (over 10 years) duration of the disease (n=25, Group B). Expectedly, **Supplementary Table A1**, significant differences were found in terms of age of onset, duration of psychotic symptoms prior to admission, and duration of antipsychotic therapy.

Significant differences were also identified in terms of the severity of the positive and general symptoms of PANSS, the severity of catatonic symptoms (BFCRS), as well as the degree of cognitive impairment (**Supplementary Table A1**) but both subgroups (A and B subgroups) did not differ substantially in the positive score (*p*=0.14). In contrast, negative symptoms and general psychopathology symptoms were more frequent in resistant SCZ patients (Subgroup B).

It is worth noting that the duration of the disease is significantly higher in subgroup B compared with subgroup A, so the prodromal period lasted about 10 years, and the duration of the disease after the manifest was at least 5 years. The duration of prodromal symptoms (11.2±6.8 years, p=0.0482) and manifesting symptoms (6.6±4.4 years, *p*=0.0003) were significantly longer in resistant patients.

Supplementary Table A1. Sociodemographic, clinical and psychometric characteristics of schizophrenic patients and healthy participants initially subdivided in two subgroups according the duration of disease and resistance to medication.

Parameter or feature		Group A (First Episode)	Group B (Resistant)	Control
Population size, n		24	25	50
Gender, n (%)	Males	12 (50)	14 (56)	19 (38)
	Females	12 (50)	11 (44)	31 (62)
Age (years) mean±SM	At start of the study	27.7±47	26.2±5.5	25.9±5.8
	At onset of prodromal symptom	20.4±6.3	15±5.1	
	At manifested syndrome	26.3±5.2	19.5±6.5	
	At onset of first psychotic symptoms	26.4±6.5	18±5.9	
	At first hospitalization	27.6±5.6	17.6±5.9	
	Duration from prodrome	7.3±5.4	11.2±6.8**	

Arthur T. Kopylov, Alexander A. Stepanov, Tatiana V. Butkova, Kristina A. Malsagova, Natalia V. Zakharova, Georgy P. Kostyuk, Artem U. Elmuratov, Anna A. Kaysheva

Parameter or feature		Group A (First Episode)	Group B (Resistant)	Control
Duration (years), mean±SM	Duration from manifestation	1.5±1.2	6.6±4.4**	
Education level, n (%)	Incomplete secondary school	0 (0)	2 (8)	1 (2)
	Secondary school	3 (13)	5 (20)	15 (30)
	Vocational school	3 (13)	10 (40)	1 (2)
	Incomplete high school	4 (16)	3 (12)	4 (8)
	High school	14 (58)	5 (20)	29 (58)
Marital status, n (%)	Married	3 (12.5)	3 (12)	15 (30)
	Singel	3 (12.5)	0 (0)	3 (6)
	Divorced	18 (75)	22 (88)	32 (64)
Occupation, n (%)	Student	6 (25)	4 (16)	37 (74)
	Employed	8 (33)	5 (20)	13 (26)
	Unemployed	10 (42)	8 (32)	0 (0)
	Disabled	0 (0)	8 (32)	0 (0)
Smoking, n (%)	Yes	5 (21)	13 (52)	11 (22)
	No	19 (79)	12 (48)	39 (78)
Hereditary loading, n (%)	Yes	15 (63)	15 (60)	
	No	9 (37)	10 (40)	
PANSS score	Total	104.2	120.8	33.1
	Positive	28.7	30.8*	8.9
	Negative	25.5	32.4	7.4
	General	50	57.6*	16.8
BFCR scale		4.8	11	0
NSA-4		18.3	23.5*	0
SAS		0.08	1.4*	0
DSM-5 score		13.2	16.5*	0
FAB score		15.3	12*	18

PANSS - Positive and Negative Syndrome Scale; BFCR scale - Bush-Francis Catatonia Rating Scale; NCS4 - the 4-Item Negative Symptom Assessment; SAS – Simpson-Angus Scale; DSM-5 - Diagnostic and Statistical Manual of mental disorders, fifth edition; FAB - Frontal Assessment Battery; * $p<0.05$ Mann-Whitney test; ** t-test, statistically significant.

Insofar principal components analysis (PCA) did not reveal meaningful differences in qualitative and quantitative items (Figure A1) in proteomes between subgroup A and subgroup B, further consideration of these two subgroups separately did not merit attention. However, a satisfied separation and good dispersion have been demonstrated between the control group merged groups of patients with SCZ.

Arthur T. Kopylov, Alexander A. Stepanov, Tatiana V. Butkova, Kristina A. Malsagova, Natalia V. Zakharova, Georgy P. Kostyuk, Artem U. Elmuratov, Anna A. Kaysheva

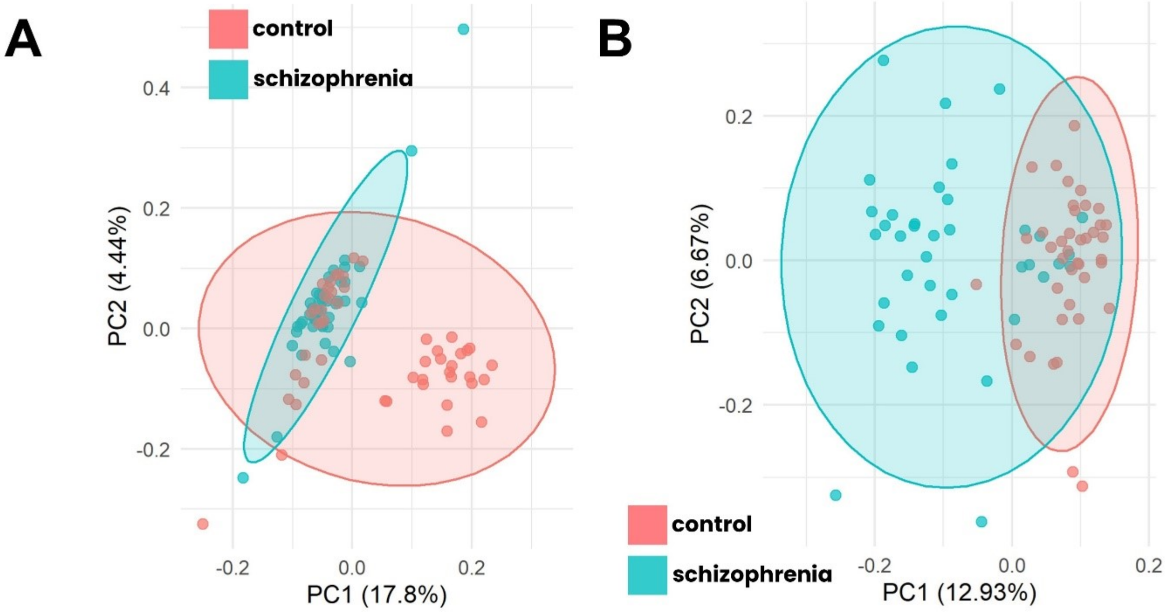

Figure A1: The designed score scattering plots show relationship between the control group and patients with schizophrenia, and the degree of variations that were explained by each component consisted of PC1=17.8% and PC2=4.44% for proteomic data (**A**) and of PC1=12.93% and PC2=6.67% for metabolomic data (**B**).

2. Subjects: combination of groups for the main consideration

By the reason of poor sensitivity of proteome to onset and duration of schizophrenia, we suggested to merge patients of the primary group (subgroups A and B) and consider them further as a single group of patients with schizophrenia irrespective of their resistance to medication. This merged cohort of patients is the main population attained further attention and consideration (**Supplementary Table A2, Study cohort)**. Additionally, we enquired patients with schizophrenia (n=28) as a validating cohort (**Supplementary Table A2**, Validating Cohort) employed for the independent validating of the measured proteins concentrations, observed metabolites ratios and overlap of the congregated proteome and metabolome between the primary (study) and validating cohort of patients with schizophrenia.

Supplementary Table A2. Sociodemographic, clinical and psychometric characteristics of schizophrenic patients after merging subgroups A and B into single Study cohort, and healthy participants.

Parameter or feature		Patients with schizophrenia		Control group	
		Study cohort	Validating cohort	Study cohort	Validating cohort
Size (n)		49	28	50	11
Gender, n (%)	Males	26 (53)	15 (53)	19 (38)	5 (45)
	Females	23 (47)	13 (47)	31 (62)	6 (55)
Age, mean+SD	Current age	26.9±5.2	28.4±7.2	25.9±5.8	26.2±4.1
	At onset of prodromal symptom	17.6±7.4	17.8±7.1	N/A	N/A
	At manifested syndrome	22.8±7.0	20.5±7.4		
	At onset of first psychotic symptoms	21.9±8.6	20.6±7.3		
	At first hospitalization	22.3±8.6	22.3±6.8		
Duration, years	Duration from prodrome	9.3±6.9	10.6±6.9	N/A	N/A
	Duration from manifestation	4.1±5.3	7.9±6.9		
Education level, n (%)	Incomplete secondary school	2 (4)	3 (11)	1 (2)	0 (0)
	Secondary school	8 (16)	7 (25)	15 (30)	2 (19)
	Vocational school	13 (27)	11 (39)	1 (2)	1 (9)
	Incomplete high school	7 (14)	2 (7)	4 (8)	1 (9)
	High school	19 (39)	5 (18)	29 (58)	7 (63)
Marital status, n (%)	Married	6 (12)	1 (3.5)	15 (30)	5 (45)
	Single	3 (6)	26 (93)	3 (6)	2 (19)
	Divorced	40 (82)	1 (3.5)	32 (64)	4 (36)
Occupation, n (%)	Student	10 (20)	6 (21)	37 (74)	5 (45)
	Employed	13 (27)	5 (18)	13 (26)	6 (55)
	Unemployed	18 (37)	17 (61)	0 (0)	0 (0)
	Disabled	8 (16)	0 (0)	0 (0)	0 (0)
Hereditary loading, n (%)	Yes	30 (61)	13 (46)	N/A	N/A
	No	19 (39)	15 (54)		
PANSS score	Total	112.5	102.7	33.1	30.7
	Positive	29.8	25.2	8.9	7.3
	Negative	28.9	31.1	7.4	6.9
	General	53.8	46.4	16.8	16.2
BFCR scale		10.5	7.9	0	0
NSA-4		21.8	20.9	0	0
SAS		1.2	0.74	0	0
DSM-5 score		14.8	14.8	0	0
FAB score		15.7	13.6	18	18

PANSS - Positive and Negative Syndrome Scale; **BFCR** scale - Bush-Francis Catatonia Rating Scale; **NCS4** - the 4-Item Negative Symptom Assessment; **SAS** – Simpson-Angus Scale; **DSM-5** - Diagnostic and Statistical Manual of mental disorders, fifth edition; **FAB** - Frontal Assessment Battery; ***** $p<0.05$ Mann-Whitney test; ****** t-test, statistically significant.

Obtained results demonstrated that a combination of the initially divided subgroups was the correct way of action. In this respect, we extracted tremendous data related to pathophysiological mechanisms of schizophrenia in the level of proteomic, metabolomic, and genome-wide associated studies. However, insufficient sensitivity of these approaches to segregate first-episode patients from those who being resistant

for treatment for a long time raised the question about either doubtfully of generally proteomic/metabolomic assays, or a kind of different divergences in the diagnosis of the certain stage of schizophrenia (i.e., first onset) which is obviously difficult to accomplish even for the expertized clinicians, or about necessity in deeper digging of proteomes and metabolomes of such patients.

We selected patients in both study and validating cohorts aligned in the majority of sociodemographic and psychometric parameters. These two groups perfectly matched in the age at onset of prodromal symptom, at manifested syndrome, at onset of first psychotic symptoms and at first hospitalization where statistical significance exceeded $p>0.83$ meaning the lack of differences between patients. The same matches were reached in regard of psychometric characteristics based on the main scales and scores routinely used in clinical psychiatry (PANSS, BFCR, NCS4 and DSM-5). The attained alignment legalized employment of the validating group to control and correct, if necessary, the hypothesized molecular mechanisms originally extracted from the proteome, metabolome and genome-wide associated studies of the assayed group of patients with schizophrenia disputed it was combined from those who had no previous history of disease and those who had been qualified as resistant to medication and therapy for a long time (more than ten years) from the manifestation.
