# Supplementary material for: Consolidation of metabolomic, proteomic, and GWAS data in connective model of schizophrenia
Source: Sci Rep. 2023 Feb 6;13:2139. doi: 10.1038/s41598-023-29117-7 (PMC9901842; doi:10.1038/s41598-023-29117-7)
Supplement: Supplementary file 2 — Supplementary Information 2. [file 41598_2023_29117_MOESM2_ESM.pdf]

Appendix B

Methods: Details of samples handling and preparations protocols for proteomic and metabolomic assays; building the calibration curve and accurate quantitation of serological markers; estimation of metabolic markers, GWAS discovery and routine statistical analysis.

Authors: Arthur T. Kopylov, Alexander A. Stepanov, Tatiana V. Butkova, Kristina A. Malsagova, Natalia V. Zakharova, Georgy P. Kostyuk, Artem U. Elmuratov, Anna A. Kaysheva

Connective model of schizophrenia: a roadmap in maze of metabolomic, proteomic and GWAS data

Arthur T. Kopylov, Alexander A. Stepanov, Tatiana V. Butkova, Kristina A. Malsagova, Natalia V. Zakharova, Georgy P. Kostyuk, Artem U. Elmuratov, Anna A. Kaysheva

Appendix B: Methods

Content

1. Reagents used throughout the workflow 1
General chemicals: 1
Enzymes: 1
Water source: 1
Solid-phase extraction: 1
Standards: 1
2. Samples preparation for proteomic assay 1
3. Liquide chromatography/high resolution mass spectrometry analysis for proteomic assay 2
4. Samples preparation for metabolomic assay 2
5. Liquide chromatography/high resolution mass spectrometry analysis for metabolomic assay 3
6. Quantitative proteomic data analysis 4
7. Qualitative proteomic data analysis 5
8. Metabolomic data analysis 5
9. Genome-wide association study 6
10. Statistical Analysis 6

1. Reagents used throughout the workflow

General chemicals:

Urea (99%) and formic acid (98%+, pure) were obtained from Acros Organics (Geel, Belgium). Trifluoroacetic acid (99%, Reagent Plus®), triethylammonium bicarbonate (1 M solution), 4-vinylpyridine (95%), sodium deoxycholic acid (>97% titration) were from Sigma (St. Louis, MO, USA). Acetonitrile (HPLC grade, filtered for 0.2 µm) was purchased from Fisher Chemical (Loughborough, UK). Acetic acid (EMSURE®, glacial, anhydrous for analysis) was from Merck (Darmstadt, Germany). TCEP (Tris(2-carboxyethyl) phosphine hydrochloride) was purchased from Pierce™ (Thermo Fisher, Rockford, IL, USA).

Enzymes:

Trypsin (sequencing grade modified), 5 vials x 20 µg, lyophilized powder (Promega; Madison, WI, USA).

β-glucuronidase from *Escherichia coli* K12 strain, 5 ml falcon vial, specific activity ~140 U/mg at 37°C (Roche Diagnostics GmbH; Germany)

Water source:

Water (TOC<3 ppb) was obtained from Milli-Q Integral 3 purification system, Millipore S.A.S (France).

Arthur T. Kopylov, Alexander A. Stepanov, Tatiana V. Butkova, Kristina A. Malsagova, Natalia V. Zakharova, Georgy P. Kostyuk, Artem U. Elmuratov, Anna A. Kaysheva

Solid-phase extraction:

Oasis® 3 cc (cubic centimeter) nominal volume, 60 mg resin, type of resin: WAX - weak anion exchange (Waters Corporation; Milford, MA, USA)

Standards:

d5-betamethasone, 1 mg, powder (catalogue number: B327002; Toronto Research Chemicals; Toronto, ON, Canada)

UPS-2 – Universal Proteomics Dynamic Range Standard, 10.6 µg, dynamic range: 0.5 fmoles – 50,000 fmoles; 48 different proteins (Sigma; Saint Louis, MO, USA)

2. Samples preparation for proteomic assay

Blood samples were collected for participating subjects in the morning between 08 am to 10 am in EDTA-2K^{+} vacuum tubes (Becton Dickinson; Franklin Lakes, NJ, USA). Totally, up to 8 mL of blood was withdrawn from each subject. Tubes were gently mixed end-over-end (approximately 10 times). Blood cells were sedimented in a pre-chilled centrifuge for 10 minutes at 2,200 *g* at 8°C. Plasma was poured off in a clean PES cryotubes and frozen immediately below -80°C until use.

The total proteins concentration was measured using a modified BCA Assay Kit (Pierce/Thermo Scientific) according manufacturer recommendations on a Nano Drop™ 2000 spectrophotometer (Thermo Scientific). The final concentrations ranged between 39 mg/mL to 52 mg/mL, median concentration was 48.2 mg/mL across the studied populations.

We used 100 µg of the measured proteins (approximately 2 µL; volume is flexible on the certain sample) for processing. Plasma samples (2 µL) were diluted in five folds (to 10 µL) by freshly prepared solution comprised of 1% deoxycholic acid sodium salt, 300 mM sodium chloride, 5 M urea, 6% acetonitrile and 15 mM tris(2-carboxyethyl)phosphine hydrochloride buffered by 75 mM triethylammonium hydrogen carbonate, pH 8.0. The average estimated final concentration of protein in a sample nearly 10 mg/mL which is appropriate for chemical reducing of cysteine amino acid residues. The reaction was performed in support with strong reducing agent stable in a wide pH range (tris(2-carboxyethyl) phosphine) and lasted for 20 minutes at 40°C with continual vigorous shaking at 700 rpm. After reaction completed, samples were chilled at ambient temperature for 3-5 minutes and proceeded for chemical alkylation for protection of the reduced cysteine residues. Alkylation was performed using a freshly prepared solution of 1% 4-vinylpyridine (stabilized) in 30% propan-2-ol (the solution is a single use only and not for storage). For this purpose, 2 µL of alkylating solution was added to samples after reducing (to 0.2% concentration finally) and the reaction mixture incubated for 30 minutes at ambient temperature (20°C-22°C) in dark place.

Plasma samples after alkylation were diluted 10 folds (to 100 µL) by 75 mM triethylammonium hydrogen carbonate solution (pH 8.0) and the estimated final concentration of proteins was nearly 1 mg/mL which is the most optimal and appropriate substrate concentration for enzymatic digestion. Digestion was performed with trypsin (modified; acetylated on lysine amino acid residues) stored in inhibiting solution of 30 mM acetic acid prior to use. Only half volume (50 µL) of the prepared sample was used for enzymatic digestion. The digestion was proceeded in two consequential steps: (1) initially 1 µg (or 5 µL) of trypsin (concentration 200 ng/µL) was added to achieve the estimated ratio to substrate as 1:50 (w/w); the reaction was incubated for 3 hours at 40°C; (2) next step, additional amount of trypsin (500 ng, or 2.5 µL) was added to achieve the final ratio to substrate as 1:100 (w/w) and the reaction was again incubated at 40°C for the next 3 hours.

The reaction (digestion) was quenched by 5 µL of 10% aqua formic acid (to 0.5% concentration finally) and centrifuged at 12,500 *g* for 10 minutes at 5°C to sediments to deoxycholic acid. The aqua layer with peptides

was collected and transferred into the new clean capillary-tubes with protected/deactivated glass to proceed high-resolution/high-accuracy mass spectrometry analysis.

3. Liquide chromatography/high resolution mass spectrometry analysis for proteomic assay

The analysis of proteome was accomplished on an ultrahigh-resolution Orbitrap Fusion mass spectrometer (Thermo Fisher; Bremen, Germany) equipped with an NSI ions source adopted for the nanoflow rate. The instrument was operated in a positive electrostatic ionization mode. The capillary voltage of system was set at -1900 V and the S-lens electrodynamic voltage was adjusted to 70% normalized value. The ion source was heated with a dry nitrogen at 240°C.

Precursor ions were surveyed and collected in C-trap within a maximum integration time of 7 ms., or until the acquisition gain control reached 3e6 ions. Ions were surveyed in a "top-speed" mode with a normalized (normalization to *m/z*=524 with z=2+) resolution of R=70K and isolated by quadrupole within ±1 Th and offset of +0.25 Th. The collected precursor ions if fallen within charge states range from z=2+ to z=5+ (ions with charge state z=1+ and uncertain charge were excluded) were triggered for the tandem MS/MS scanning.

Triggered ions were decomposed in a linear ions-trap (LIT) and fragmentation was supplied by a mix of helium/nitrogen collision gas (25%/75%). Fragment ions were obtained in HCD (high-energy collision dissociation) mode after applying the normalized activation energy (27% normalization) in a stepwise manner (ramping within ±20%).

Peptides of the digested plasma samples were separated on an Ultimate 3000 RSLC UPLC system (Thermo Fisher; Bremen, Germany) equipped with loading microflow and analytical binary pumps. Sample (2 µL, or estimated amount of 1 µg) were loaded onto the enrichment pre-analytical column Acclaim Pepmap® (5 mm x 0.3 mm, 300 A pore size, 5 µm particle size) at a flow rate of 15 µL/min for 6 minutes in an isocratic mode of the mobile phase C comprised of water supplemented with 2.5% acetonitrile, 0.03% acetic acid and 0.01% formic acid (pH=2.63-2.65 at temperature of 20.5°C). After sample has been loaded, the divert valve switched to position for elution and separation of peptide onto analytical column Acclaim Pepmap® (75 µm x 150 mm, 1.8 µm particle size, 60 A pore size) at 0.3 µL/min flow rate.

Peptides were separated in a gradient of mobile phase A (water with 0.03% acetic acid and 0.01% formic acid) and mobile phase B (80% acetonitrile, 20% methanol, 0.03% acetic acid and 0.01% formic acid). The following elution scheme was applied (normalized to the content of mobile phase B (m. p. B)): 0 – 7 minutes: 2.5% of m. p. B; 7 – 27 minutes: 2.5% - 13% of m. p. B; 27 – 38 minutes: 13% – 28% of m. p. B; 38 – 45 minutes: 28% - 32% of m. p. B; 45 – 51 minutes: 28% - 30% m. p. B; 51 – 53 minutes: 30% - 90% of m. p. B and rapid increasing the flow rate to 0.45 µL/min; 53 – 60 minutes: isocratic 90% of m. p. B at 0.45 µL/min; 60 – 63 minutes: 90% - 2.5% of m. p. B with retarding the flow rate to 0.3 µL/min; 63 – 76 minutes – 2.5% of m. p. B. At 65 minutes the divert valve was switched to "loading" position to accelerate equilibration of enrichment pre-column in the mobile phase C (water supplemented with 2.5% acetonitrile, 0.03% acetic acid and 0.01% formic acid).

4. Samples preparation for metabolomic assay

Total metabolic fractions were extracted from exactly the same samples used for the proteomic assay. For this purpose, 100 µL of plasma obtained as a rest of blood handling as described in *"1.Samples preparation for proteomic assay"* was fortified with 10 µL of deuterated d5-betamethasone in methanol (catalogue number: B327002, Toronto Research Chemicals; Toronto, ON, Canada) used as an internal standard to 100

ng/mL finally. Plasma was diluted with ice-cold methanol to 400 µL for deproteinization. Samples were vigorously stirred for 10 minutes at 15°C and then centrifuged at 10°C for 15 minutes at acceleration of 10,000 *g* to sediment precipitated proteins. Pellet was discarded, and the supernatant quantitatively (300 µL) was transferred to a new clean glass tube. The solution of deproteinized plasma was diluted to 3 mL with 50 mM sodium phosphate buffer, pH 6.3. Then, the obtained solution was fortified with 50 µL of β-glucuronidase from *Escherichia coli* K12 strain (Roche Diagnostics GmbH; Germany) was added to the buffered solution and incubated for 1 hour at 55°C. The hydrolysis of glucuronic conjugates was quenched with 15 mM of potassium carbonate, pH 9.0. Then, for LLE (liquid-liquid extraction) extraction 1.5 mL of ethylacetate with n-pentane (90:10) was added to samples and vigorously shacked for 5 minutes at 20°C then let it stand at -20°C for 15 minutes; the organic layer was transferred, and the extraction procedure was repeated. The collected fractions were combined and dried under nitrogen flow.

The aqueous phase was transferred to WAX (weak anion exchange) SPE (solid-phase extraction) cartridge (3 cc, 60 mg, Oasis™ series, Waters) preconditioned with 3 mL of 2% hydroxide ammonia solution and 2 x 3 mL of water. On-loaded metabolic compounds were consequently washed with 3 mL of water and eluted with 2 x 3 mL of 2% formic acid and 3 mL of methanol. The eluted fractions were combined and dried under vacuum at 45°C.

Residues after SPE and LLE were reconstituted in 100 µL of aqueous acetonitrile (50:50, v/v) shacked for 10 minutes at ambient temperature, centrifuged for 10 minutes at 15oC and the supernatant was quantitatively (80 µL) transferred to a new clean capillary from deactivated glass for the high-resolution LC-MS analysis.

5. Liquide chromatography/high resolution mass spectrometry analysis for metabolomic assay

The analysis of total metabolome fraction was conducted on a high-resolution quadrupole time-of-flight (Q-TOF) G6550A mass spectrometer (Agilent, Inc.; Waldbronn, Germany) equipped with Dual Jet-Stream ionization source (Agilent Inc.; Waldbronn, Germany) and coupled with 1290 Infinity ultra-high performance liquid chromatography (UPLC) system (Agilent Inc.; Waldbronn, Germany) composed of autosampler with pre-installed 20 µL loop, column thermostat, autosampler thermostat, binary pump and UV diode array detector.

The instrument (mass spectrometer) was operated in a positive ionization mode, in 4 GHz high resolution mode with a slicer index position set to “6 of 9” (high-resolution mode). The instrument was tuned in a range of calibrating masses up to 1700 *m/z* and the nominal resolution exceeded R>46K at a mass of *m/z*=922. A high-pressure ion funnel DC voltage was adjusted to 90 V, whereas a low-pressure funnel DC voltage was set at 55 V. The fragmentor voltage was set to 320 V when tuning the mass spectrometer. The capillary voltage was adjusted to -3700 V and the nozzle voltage was set to -1400 V. Drying gas (nitrogen) with a flow rate of 14 L/min was heated at 280°C and sheath gas (nitrogen) at a flow rate of 11 L/min was heated at 240°C, while the nebulizer pressure was optimized for the LC flow rate (0.4 mL/min) and adjusted to 21 psi (or 1.45 bar).

Precursor ions were surveyed in a range of 100-1000 *m/z* with accumulation time of 150 ms per one transition. Top five precursor ions with intensity exceeded 3000 counts (*cnts*) and priority charge states z=1+ or z=2+ were selected to pass quadrupole mass filter and triggered to fragmentation. Fragmentation was accomplished in a CID (collision-induced dissociation) with support of nitrogen as a collision gas at adjusted pressure 25 psi (or 1.72 bar). Fragment ions were scanned within a range of 100-1000 *m/z* with intensity cut-off level of 250 counts (*cnts*). Dynamic accumulation of at least 19,500 counts/transition and 6 scans per a spectrum were set. Ions were detected by and MCP time-of-flight mass detector with adjusted voltage of 710 V. The complete one duty cycle was estimated as 1,250 sec.

Chromatography separation was performed on an Acclaim™ Phenyl-1 column (2.1 x 150 mm, 3 µm particle size, 120 A pore size; Thermo Scientific) regularly heated at 45°C. The elution was conducted at a flow rate of 0.3 mL/min in a gradient of mobile phase A (water supplied with 0.01% formic acid, 0.0085% heptafluorobutyric acid) and mobile phase B (30% methanol, 70% acetonitrile, 0.01% formic acid and 0.0085% heptafluorobutyric acid). The starting condition of elution gradient (normalized to mobile phase B, m. p. B) was 7.5% of B and the following eluting scheme was applied: 0 – 1.8 minutes: 7.5% of m. p. B; 1.8 – 5.5 minutes: 7.5% - 20% of m. p. B; 5.5 – 7.0 minutes – 20% of m. p. B; 7.0 – 18 minutes: 20% - 47% of m. p. B; 18 – 20 minutes: 47% - 50% of m. p. B; 20 – 23 minutes: 50% of m. p. B; 23 – 27 minutes: 50% - 62% of m. p. B; 27 – 29 minutes: 62% - 90% of m. p. B; 29.0 – 29.5 minutes – 90% - 95% of m. p. B; 29.5 – 33.5 minutes – 95% of m. p. B; 33.5 – 34.0 minutes: 95% - 20% of m. p. B; 34.0 – 35.5 minutes: 20% of m. p. B; 35.5 – 36.0 minutes – 20% - 95% of m. p. B; 36 – 40 minutes: 95% of m. p. B; 40 – 41 minutes: 95% - 7.5% of m. p. B; post-analysis column equilibration in initial gradient condition of 7.5% of m. p. B was lasted for 10 minutes at a flow rate of 0.3 mL/min before the next sample run.

6. Quantitative proteomic data analysis

We used Dynamic Universal Proteomic Standard UPS-2™ (Sigma; Saint Louis, MO, USA) specifically designed and developed on 2006 year in collaboration with the Association of Biomolecular Resource Facilities (ABRF) for the large-scale proteomic quantitative analysis. The UPS-2™ comprises of 48 different proteins ranged in amounts between 0.5 fmoles to 50,000 fmoles in a single tube. The total content of tube is 10.6 µg. Aspect information about the proteins listed in the UPS-2™, including their sequences, amount in the tube, possible post-translational modifications and sources, is accessible in the following link: http://www.sigma.com/ups

Calibration was accomplished using a set of UPS-2™ standard which has been spiked into matrix to the final concentration the targeted proteins 0.025 fmoles/µL to 2500 fmoles/µL, while the total concentration of matrix proteins was estimated as 500 ng/µL and a volume of 2µL (or 1 µg) of the fortified matrix has been taken for the analysis. Matrix solution was prepared from the non-human proteins, thence, initially 1e6 cells of *Escherichia coli* (strain *K12*) was chosen for this purpose to avoid possible interference with (a) endogenous human proteins and (b) customized UPS-2™ proteins. The matrix was prepared exactly in the same way as described for the assayed samples including type of the selected alkylation by 4-vinylpyridine. Analysis was performed on a high-resolution mass spectrometer as described for the assayed samples in the section "*Liquide chromatography/high resolution mass spectrometry analysis for proteomic assay*". Matrix sample fortified with the UPS-2™ standard was analyzed in 10 replicates and the analysis was completely repeated on another UPS-2™ tube to estimate accuracy and reproducibility.

Obtained raw mass spectrometry data were analyzed using Max Quant software (version 1.6.13) **[1]** with support of proprietary FASTA-file appropriate for the UPS-2™ standard provided by vendor. The protein was accounted as confidently detected if it recognized by at least three different unique peptides and mass error did not exceed 5 ppm (±2.5 ppm) for the unmodified peptide and 3 ppm (±1.5 ppm) for the modified counterpart (if applicable). Only peptides with complete cleavage were considered (no missed cleavages were allowed) and maximum of two fixed/flexible modifications were allowed. Peptide should be covered on at least 70% by fragmentation spectra and mass error of the detected fragments should not exceed 0.005 Da (±0.0025 Da). The protein was included in the calibration plot if detected in at least 6 replicates of 10 and the standard deviation of estimated iBAQ (intensity-based absolute quantification approach) value within calibration point did not exceed 20%. Linear regression model was considered for the calibration plot; since the dynamic range of concentration covers five orders of magnitude, weighting factor of 1/x was applied to fit calibrating points with linear regression.

Arthur T. Kopylov, Alexander A. Stepanov, Tatiana V. Butkova, Kristina A. Malsagova, Natalia V. Zakharova, Georgy P. Kostyuk, Artem U. Elmuratov, Anna A. Kaysheva

7. Qualitative proteomic data analysis

Acceptance criteria for the protein identification were based on the Human Proteome Project Mass Spectrometry Data Interpretation Guidelines 3.0 but were applied in more flexible manner. At least two distinct by sequence peptides of which one must be unique (proteotypic) peptide (apart of isoform-specific peptides) were accepted for regular proteins identification. Data analysis was performed in the Max Quant environment (version 1.6.13). Three-dimensional peaks (intensity, *m/z* value and retention time) were detected for the data-dependent analysis. The total number of fragment ions per 100 *m/z* scale was limited to 20 for the high complexity protein mixtures. Estimated precursor ion masses were associated with probable fragment ion masses on the basis of a cosine correlation value of at least 0.8.

Search was performed in Max Quant against the human indexing database for amino acid sequences of proteins obtained as a FASTA-file from the UniProt KB (release March 2020 (26,549 reviewed entries) to which concatenated decoy sequences in a reverse fashion were added. For calibrating purposes, a small database consisting only of the UPS-2™ proteins as described above in subsection "*5. Quantitative proteomic data analysis*". The search was performed with an initial precursor mass tolerance of 5 ppm and fragment mass tolerance of 0.001 Da. We included *S*-pyridilethylation, as a fixed modification and oxidized methionine and Q/N deamidation as variable modifications. The minimal peptide length was fixed to 8 amino acids, and we allowed up to one missed cleavage.

For peptide and protein identifications, the 1% false discovery rate (FDR) was determined by accumulating the reverse database hits. If the identified peptide was not undoubtedly linked to a specific protein isoform, all isoforms were grouped as one protein. To avoid apparent misidentifications that are only due to protein name discrepancies, gene names and UniProt IDs were manually examined.

8. Metabolomic data analysis

Metabolomic data analysis was conducted in the Mass Hunter Qualitative Analysis (Agilent Inc., version B05) and Mass Hunter Metabolite ID (Agilent Inc., version) with the pre-installed integrated METLIN Personal Metabolite Compound Database (release 2017). Searching and extraction of compounds from raw data (Agilent proprietary "d"-file-folder) was performed according the following criteria: mass range restriction of 100-1000 m/z; charge state restriction up to z=2+ and allowed dimers fit to ion formula $[2M+H]^+$; retention time was restricted to 0.2-28.7 minutes. The following types of ions and adduct were interrogated across the chromatographic profile: $[M+H]^+$, $[M+2H]^{2+}$, $[M+Na]^+$, $[M+K]^+$, $[M+CH3COO]^+$. Mass accuracy was evaluated as asymmetric mass shift of ±20 ppm and +0.015 mDa per each 100 *m/z* units; Kendrick mass error was skipped. Extraction of compounds with isotopies distribution succumb the common organic molecules (within 25% window) was prioritized while ions with isotopes envelope corresponding halogenated molecules or envelopes with harmonic repetition were unprioritized. Preliminary only ions with total score of at least 70 arbitrary units (of 100) were populated. Extracted compounds were surveyed against the METLIN database to attribute metabolite or their combination and only compounds with total final score of at least 85 after revision were survived. Conflict interfering masses (compounds) were resolved according their appearance along the chromatographic profile (match between different patients at a retention time, m/z value), mass error toward the theoretical mass (not exceeding the window of 10 ppm), presence of witness salt-adducts (sodium and/or potassium adducts), fit to the fragmentation spectra in the customized spectra library (in METLIN and in support with spectra library of Scripps Centre for Metabolomics, accessed on https://xcmsonline.scripps.edu/landing_page.php?pgcontent=mainPage). Endogenous human specific metabolites were enriched against the HMDB (Human Metabolites Database) database which has been

downloaded as XML-library. Clinically relevant drug metabolites were filtered through HMDB and DB (Drug Bank) databases and were excluded for the consideration.

9. Genome-wide association study

Total DNA isolation was carried out using QIAamp DNA Kit (Qiagen, Hilden, Germany). Case (48 patients with schizophrenia) and control “1000 Genomes Phase 3” (200 volunteers) are taken from the same European population. Sample preparation and scanning of chips on an Illumina HiScan instrument (San Diego, CA, USA). Sample genotyping for 652,297 markers were performed using Infinium Global Chips Screening Array-24 version 2.0. Genotyping was carried out using the IMPUTE2 program with parameters Ne = 20,000 and k = 90. After imputing, additional filtering was performed by metrics info> 0.5 and genotype probability> 0.5. Genetic marker frequencies programming language *R*. Quality Control and Association Search was conducted using the plink version 1.90b6.7 program. Control of the homogeneity of the obtained sample of samples was carried out using a PCA made by smart-PCA version13050 EIGENSOFT package.

10. Statistical Analysis

Significance of anthropometric and psychometric items between the studied groups was performed by two-sided *t*-test at a *p*-value cut-off p<0.05. Due to the small size of the studied groups, significance in the measured concentrations of proteins and metabolites was evaluated by Fisher's exact test with significance level cut-off *p*<0.05. Principal component analysis (PCA) was applied to the total proteome and metabolome identified in both control and assayed (subjects with schizophrenia) groups. To performed PCA, a tentative percolation of proteins was appreciated. To achieve data reduction and discrimination, principal component analysis (PCA) and pairwise Student's t-test at p<0.05 were applied to the total proteome and metabolome identified in both control and assayed (subjects with schizophrenia) groups. Alternatively, to perform variable selection and classification of the studied cohort, sparce partial least-squares discriminant analysis (sPLS-DA) with 0.95 ellipse area confidence level was utilized. To be selected from the total proteome, the candidate protein shall meet the criterion of unicity when more than one (n_i(p)>1) unique peptide shall be met for the certain protein among the totality of peptides. NSAFs (Normalized Spectra Abundancy Factor) were summarized for each protein within the studied groups and data with zero means were imputated. For metabolites the criteria of frequency exceeding one occasion within each studied group was significant. The significance scores obtained after identification of metabolite compounds (see section “*8. Metabolomic data analysis*”) were averaged within each studied group, and zero means were imputated. The selected proteins and metabolites meaningfully separating subjects of the control group from those in group with schizophrenia were applied for Wilcox test and estimated *p*-values were plotted on Volcano against calculated fold changes. Proteins and metabolites, significantly altered between the studied groups, were extracted and submitted for functional and pathways annotation analysis at a *q*-value threshold less than 0.01 using Gene Ontology (GO) toolset [2] against complete human genome as a reference list. The enriched terms were refined with similarity coefficient of >0.7 in way to remove the redundant terms. The refined terms were associated with the protein and metabolite lists drawn up for the studied groups, and adjusted with KEGG [3] means and Reactome [4] pathway terms. All other analyses were performed in-house scripts written in R (R Foundation for Statistical Computing, Vienna, Austria) [5].

[1] Wichmann C, Meier F, Virreira Winter S, Brunner AD, Cox J, Mann M. MaxQuant.Live Enables Global Targeting of More Than 25,000 Peptides. Mol Cell Proteomics. 2019;18(5):982-994. doi:10.1074/mcp.TIR118.001131

[2] Ashburner M, Ball CA, Blake JA, et al. Gene ontology: tool for the unification of biology. The Gene Ontology Consortium. Nat Genet. 2000;25(1):25-29. doi:10.1038/75556

[3] Kanehisa M, Sato Y. KEGG Mapper for inferring cellular functions from protein sequences. Protein Sci. 2020;29(1):28-35. doi:10.1002/pro.3711

[4] Fabregat A, Jupe S, Matthews L, et al. The Reactome Pathway Knowledgebase. Nucleic Acids Res. 2018;46(D1):D649-D655. doi:10.1093/nar/gkx1132

[5] R Development Core Team. R: A Language and Environment for Statistical Computing. Available online: https://www.gbif.org/zh/tool/81287/r-a-language-and-environment-for-statistical-computing
