# Supplementary material for: Consolidation of metabolomic, proteomic, and GWAS data in connective model of schizophrenia
Source: Sci Rep. 2023 Feb 6;13:2139. doi: 10.1038/s41598-023-29117-7 (PMC9901842; doi:10.1038/s41598-023-29117-7)
Supplement: Supplementary file 3 — Supplementary Information 3. [file 41598_2023_29117_MOESM3_ESM.pdf]

Appendix C

Proteome: complete list of proteins shared between the group of patients with schizophrenia and healthy donors (control group). Relative ratios (estimated as fold-changes, FC) was calculated for all proteins, but the absolute concentrations were calculated only for proteins with strong segregation ability according the PCA test results.

The mass spectrometry proteomics data have been deposited to the ProteomeXchange Consortium via the PRIDE partner repository with the dataset identifier **PXD035863** and 10.6019/PXD035863

Authors: Arthur T. Kopylov, Alexander A. Stepanov, Tatiana V. Butkova, Kristina A. Malsagova, Natalia V. Zakharova, Georgy P. Kostyuk, Artem U. Elmuratov, Anna A. Kaysheva

Connective model of molecular events associated with schizophrenia pathogenesis

Arthur T. Kopylov, Alexander A. Stepanov, Tatiana V. Butkova, Kristina A. Malsagova, Natalia V. Zakharova, Georgy P. Kostyuk, Artem U. Elmuratov, Anna A. Kaysheva

Appendix C: Proteome

Content

1. Shared proteome and meaningful proteins with discriminating ability 1
2. Proteins interaction........6

1. Shared proteome and meaningful proteins with discriminating ability

The mass spectrometry proteomics data have been deposited to the ProteomeXchange Consortium via the PRIDE partner repository with the dataset identifier PXD035863 and 10.6019/PXD035863

The contrast analysis of proteomes revealed n=159 unique identified proteins commonly distributed among groups of patients with schizophrenia and healthy donors (control group). Based on the obtained semi-quantitative data expressed in NSAF units (Normalized Spectral Abundance Factor) [**1**,**2**,**3**,**4**], we estimated fold-changes (FC, or relative ratio) of the protein representation in group of patients with schizophrenia toward the group of healthy donors. When relative ratios (FC) were calculated, the frequency of protein in each studied group separately was accounted. Since the frequency for some proteins can be insufficiently low, we calculated fold-changes as mean and as median.

The collected proteins were tested using PCA analysis and a minor fraction (n=24) delivered satisfied ability to segregate patients with schizophrenia from those who never had a history of this psychopathology (statistical significance at adjusted p-value cut-off $p<0.05$ using Mann-Whitney test). These proteins were extracted from the pool of shared proteome (**Supplementary Table B1**; color-filled rows) and applied on the plotted calibration curve (see **Supplementary Methods** for details of calibration) for the accurate absolute quantitative determination (see **Table 1 in the Main Paper**).

Supplementary Table B1. Complete list of proteins (n=159) shared between the control group of healthy volunteers and patients with schizophrenia (see Supplementary Table A2 in the Appendix-A). Fold changes were calculated for all elements of the shared proteome but only small fraction of proteins (n=24; color-filled with blue) demonstrated ability to discriminated patients with schizophrenia from healthy donors (see Table 1 in the Main Paper). Proteins are ordered according their representation (fold changes, FC) in patients with schizophrenia compared to healthy donors (control group).

Main Accession	Primary Gene name	Protein names	MW, kDa	Freq. in SZC group	Freq. in control group	FC (NSAF), mean	FC (NSAF), median	Log2
P0CG04	IGLC1	Immunoglobulin lambda constant 1	11.34	0.04	0.18	0.28	0.28	-1.83
P43652	AFM	Afamin	69.02	0.78	0.96	0.44	0.41	-1.30
P01019	AGT	Angiotensinogen	53.12	0.82	0.84	0.67	0.49	-1.02
P02750	LRG1	Leucine-rich alpha-2-glycoprotein	38.15	0.6	0.54	0.54	0.49	-1.02
O00512	BCL9	B-cell CLL/lymphoma 9 protein	149.19	0.04	0.02	0.50	0.50	-1.00
P01709	IGLV2-8	Immunoglobulin lambda variable 2-8	12.37	0.16	0.1	0.75	0.50	-1.00
P02743	APCS	Serum amyloid P-component	25.37	0.02	0.16	0.42	0.50	-1.00
Q99459	CDC5L	Cell division cycle 5-like protein	92.19	0.06	0.02	0.50	0.50	-1.00
P00736	C1R	Complement C1r subcomponent	80.07	0.56	0.56	0.53	0.51	-0.98
P04264	KRT1	Keratin, type II cytoskeletal 1	66.00	0.1	0.38	0.58	0.55	-0.86
P01834	IGKC	Immunoglobulin kappa constant	11.76	1	0.98	0.45	0.56	-0.83

Main Accession	Primary Gene name	Protein names	MW, kDa	Freq. in SZC group	Freq. in control group	FC (NSAF), mean	FC (NSAF), median	Log2
P06727	APOA4	Apolipoprotein A-IV	45.37	0.98	0.96	0.63	0.58	-0.79
P02652	APOA2	Apolipoprotein A-II	11.17	1	0.98	0.49	0.58	-0.78
P01614	IGKV2D-40	Immunoglobulin kappa variable 2D-40	13.30	0.02	0.02	0.58	0.58	-0.78
Q96PD5	PGLYRP2	N-acetylmuramoyl-L-alanine amidase	62.18	0.98	0.82	0.72	0.59	-0.77
P51884	LUM	Lumican	38.40	0.36	0.48	0.65	0.60	-0.74
P07996	THBS1	Thrombospondin-1	129.30	0.8	0.8	0.82	0.62	-0.70
P01619	IGKV3-20	Immunoglobulin kappa variable 3-20	12.55	0.82	0.88	0.67	0.63	-0.68
P04433	IGKV3-11	Immunoglobulin kappa variable 3-11	12.57	0.36	0.22	0.64	0.64	-0.65
P07357	C8A	Complement component C8 alpha chain	65.12	0.78	0.8	0.69	0.64	-0.64
P02747	C1QC	Complement C1q subcomponent subunit C	25.76	0.9	0.72	0.72	0.65	-0.63
P02749	APOH	Beta-2-glycoprotein 1	38.27	1	0.98	0.64	0.66	-0.61
P29622	SERPINA4	Kallistatin	48.51	0.14	0.46	0.69	0.66	-0.61
O75636	FCN3	Ficolin-3	32.88	0.06	0.22	0.67	0.67	-0.58
P02655	APOC2	Apolipoprotein C-II	11.28	0.92	0.82	0.75	0.67	-0.58
P22792	CPN2	Carboxypeptidase N subunit 2	60.52	0.06	0.34	0.69	0.67	-0.58
Q8TDX9	PKD1L1	Polycystic kidney disease protein 1-like 1	315.23	0.02	0.04	0.67	0.67	-0.58
P00915	CA1	Carbonic anhydrase 1	28.85	0.08	0.02	0.75	0.67	-0.58
P04217	A1BG	Alpha-1B-glycoprotein	54.22	0.98	0.96	0.76	0.68	-0.55
P0C0L5	C4B; C4B_2	Complement C4-B	192.63	0.22	0.36	0.69	0.68	-0.55
P04004	VTN	Vitronectin	54.27	0.98	0.96	0.68	0.69	-0.54
P02765	AHSG	Alpha-2-HS-glycoprotein	39.30	1	1	0.70	0.69	-0.54
P02656	APOC3	Apolipoprotein C-III	10.85	0.94	0.92	0.64	0.69	-0.53
P13671	C6	Complement component C6	104.72	0.46	0.6	0.60	0.70	-0.52
P19827	ITIH1	Inter-alpha-trypsin inhibitor heavy chain H1	101.33	1	0.98	0.69	0.70	-0.52
P08185	SERPINA6	Corticosteroid-binding globulin	45.11	0.22	0.48	0.68	0.71	-0.50
P02768	ALB	Serum albumin	69.32	1	1	0.65	0.71	-0.49
P04114	APOB	Apolipoprotein B-100	515.28	1	0.98	0.82	0.71	-0.49
P08697	SERPINF2	Alpha-2-antiplasmin	54.53	0.7	0.82	0.64	0.71	-0.49
P05546	SERPIND1	Heparin cofactor 2	57.03	0.74	0.96	0.70	0.72	-0.48
P01009	SERPINA1	Alpha-1-antitrypsin	46.71	1	1	0.67	0.72	-0.48
P35858	IGFALS	Insulin-like growth factor-binding protein complex acid labile subunit	65.99	0.89	0.62	0.85	0.72	-0.47
O95445	APOM	Apolipoprotein M	21.24	0.1	0.44	0.68	0.72	-0.47
P35527	KRT9	Keratin, type I cytoskeletal 9	62.03	0.04	0.2	0.70	0.72	-0.47
P02790	HPX	Hemopexin	51.64	1	0.98	0.74	0.73	-0.46
P02766	TTR	Transthyretin	15.88	0.88	0.86	0.80	0.73	-0.45
P01857	IGHG1	Immunoglobulin heavy constant gamma 1	36.08	1	1	0.72	0.74	-0.43
A0A0B4J1V1	IGHV3-21	Immunoglobulin heavy variable 3-21	12.83	0.4	0.24	0.81	0.75	-0.42
P00747	PLG	Plasminogen	90.51	1	0.98	0.76	0.75	-0.41
P0C0L4	C4A	Complement C4-A	192.66	0.46	0.26	0.83	0.76	-0.40
P02654	APOC1	Apolipoprotein C-I	9.33	0.68	0.62	0.73	0.76	-0.40
P05155	SERPING1	Plasma protease C1 inhibitor	55.12	0.98	0.94	0.78	0.76	-0.39
P01871	IGHM	Immunoglobulin heavy constant mu	49.41	1	0.98	0.73	0.76	-0.39
P01877	IGHA2	Immunoglobulin heavy constant alpha 2	36.57	0.4	0.4	0.81	0.77	-0.38
P02763	ORM1	Alpha-1-acid glycoprotein 1	23.50	1	0.98	0.81	0.78	-0.37
P06396	GSN	Gelsolin	85.64	0.98	0.94	0.78	0.78	-0.36
P36955	SERPINF1	Pigment epithelium-derived factor	46.28	0.18	0.58	0.70	0.79	-0.35
P02774	GC	Vitamin D-binding protein	52.93	1	0.98	0.73	0.80	-0.33
P27918	CFP	Properdin	51.24	0.88	0.8	0.89	0.80	-0.32
P00748	F12	Coagulation factor XII	67.75	0.22	0.56	0.86	0.80	-0.32
P00450	CP	Ceruloplasmin	122.13	1	0.98	0.79	0.80	-0.32
P06681	C2	Complement C2	83.21	0.72	0.42	0.82	0.82	-0.29
P02746	C1QB	Complement C1q subcomponent subunit B	26.70	0.78	0.64	0.83	0.82	-0.29
P01768	IGHV3-30	Immunoglobulin heavy variable 3-30	12.94	0.02	0.16	0.92	0.82	-0.28
P02753	RBP4	Retinol-binding protein 4	23.00	0.98	0.94	0.81	0.83	-0.27

Main Accession	Primary Gene name	Protein names	MW, kDa	Freq. in SZC group	Freq. in control group	FC (NSAF), mean	FC (NSAF), median	Log2
P00734	F2	Prothrombin	69.99	0.98	0.98	0.85	0.83	-0.27
Q14624	ITIH4	Inter-alpha-trypsin inhibitor heavy chain H4	103.29	0.98	0.98	0.80	0.83	-0.27
P01593	IGKV1D-33	Immunoglobulin kappa variable 1D-33	12.84	0.06	0.1	1.03	0.83	-0.26
O14791	APOL1	Apolipoprotein L1	43.95	0.36	0.48	0.80	0.83	-0.26
P02745	C1QA	Complement C1q subcomponent subunit A	26.00	0.04	0.36	0.75	0.83	-0.26
P02748	C9	Complement component C9	63.13	0.98	0.96	0.90	0.84	-0.26
P01023	A2M	Alpha-2-macroglobulin	163.19	1	1	0.86	0.84	-0.25
P19823	ITIH2	Inter-alpha-trypsin inhibitor heavy chain H2	106.40	0.98	0.98	0.83	0.85	-0.24
P02751	FN1	Fibronectin	262.46	0.98	0.96	0.86	0.85	-0.23
P01024	C3	Complement C3	187.03	1	1	0.90	0.86	-0.22
P01611	IGKV1D-12	Immunoglobulin kappa variable 1D-12	12.61	0.1	0.14	0.84	0.86	-0.22
P02787	TF	Serotransferrin	77.01	1	1	0.89	0.86	-0.21
P20851	C4BPB	C4b-binding protein beta chain	28.34	0.04	0.26	0.86	0.88	-0.19
A0A0J9YX35	IGHV3-64D	Immunoglobulin heavy variable 3-64D	12.81	0.06	0.02	0.77	0.88	-0.19
P03952	KLKB1	Plasma kallikrein	71.32	0.46	0.8	0.91	0.88	-0.18
P02760	AMBP	Protein AMBP	38.97	0.98	0.98	0.88	0.88	-0.18
P06312	IGKV4-1	Immunoglobulin kappa variable 4-1	13.37	0.44	0.58	0.89	0.89	-0.17
P00738	HP	Haptoglobin	45.18	1	0.98	0.92	0.89	-0.17
P69905	HBA1; HBA2	Hemoglobin subunit alpha	15.25	0.9	0.8	0.97	0.89	-0.17
P01782	IGHV3-9	Immunoglobulin heavy variable 3-9	12.94	0.02	0.12	0.84	0.89	-0.17
A0A0C4DH38	IGHV5-51	Immunoglobulin heavy variable 5-51	12.67	0.04	0.06	0.89	0.89	-0.17
A0A0C4DH42	IGHV3-66	Immunoglobulin heavy variable 3-66	12.69	0.04	0.06	0.81	0.89	-0.16
P08519	LPA	Apolipoprotein))	501.00	0.32	0.36	1.44	0.90	-0.15
P05090	APOD	Apolipoprotein D	21.26	0.98	0.94	0.92	0.90	-0.15
Q14512	FGFBP1	Fibroblast growth factor-binding protein 1	26.25	0.24	0.26	0.98	0.90	-0.15
P01008	SERPINC1	Antithrombin-III	52.57	0.98	0.98	0.93	0.90	-0.15
P27169	PON1	Serum paraoxonase/arylesterase 1	39.71	0.82	0.96	0.89	0.90	-0.15
P01861	IGHG4	Immunoglobulin heavy constant gamma 4	35.92	0.9	0.98	0.82	0.90	-0.15
P02649	APOE	Apolipoprotein E	36.13	0.96	0.94	0.90	0.91	-0.14
P02647	APOA1	Apolipoprotein A-I	30.76	1	1	0.94	0.92	-0.13
O43866	CD5L	CD5 antigen-like	38.06	0.74	0.82	0.87	0.92	-0.11
P01615	IGKV2D-28	Immunoglobulin kappa variable 2D-28	12.95	0.4	0.74	0.89	0.92	-0.11
A0A0C4DH67	IGKV1-8	Immunoglobulin kappa variable 1-8	12.53	0.04	0.04	0.93	0.93	-0.11
P01597	IGKV1-39	Immunoglobulin kappa variable 1-39	12.73	0.08	0.06	1.02	0.93	-0.10
P01042	KNG1	Kininogen-1	71.91	1	0.98	0.97	0.93	-0.10
P01700	IGLV1-47	Immunoglobulin lambda variable 1-47	12.28	0.96	0.82	0.94	0.94	-0.09
P01876	IGHA1	Immunoglobulin heavy constant alpha 1	37.63	1	0.98	0.96	0.94	-0.09
P01591	JCHAIN	Immunoglobulin J chain	18.09	0.98	0.86	0.91	0.94	-0.08
P01031	C5	Complement C5	188.19	1	0.96	0.99	0.95	-0.07
P07225	PROS1	Vitamin K-dependent protein S	75.07	0.48	0.82	0.97	0.96	-0.06
P10643	C7	Complement component C7	93.46	0.9	0.74	1.02	0.96	-0.06
P01011	SERPINA3	Alpha-1-antichymotrypsin	47.62	1	0.98	0.89	0.96	-0.06
P01859	IGHG2	Immunoglobulin heavy constant gamma 2	35.88	0.98	1	1.01	0.97	-0.05
P10909	CLU	Clusterin	52.46	0.98	0.96	1.02	0.98	-0.03
P20742	PZP	Pregnancy zone protein	163.76	0.04	0.02	0.98	0.98	-0.02
P08603	CFH	Complement factor H	139.00	1	0.98	0.96	0.99	-0.02
A0A075B6S6	IGKV2D-30	Immunoglobulin kappa variable 2D-30	13.21	0.06	0.02	0.89	1.00	0.00
A0A0B4J2D9	IGKV1D-13	Immunoglobulin kappa variable 1D-13	12.56	0.02	0.02	1.00	1.00	0.00
O75818	RPP40	Ribonuclease P protein subunit p40	41.81	0.02	0.02	1.00	1.00	0.00
O75896	TUSC2	Tumor suppressor candidate 2	12.07	0.08	0.2	1.15	1.00	0.00
P01880	IGHD	Immunoglobulin heavy constant delta	42.33	0.22	0.38	0.84	1.00	0.00
P02775	PPBP	Platelet basic protein	13.89	0.16	0.54	0.99	1.00	0.00
P02776	PF4	Platelet factor 4	10.84	0.18	0.18	1.02	1.00	0.00
P05452	CLEC3B	Tetranectin	22.52	0.16	0.28	1.11	1.00	0.00
P07358	C8B	Complement component C8 beta chain	67.00	0.96	0.88	1.08	1.00	0.00

Main Accession	Primary Gene name	Protein names	MW, kDa	Freq. in SZC group	Freq. in control group	FC (NSAF), mean	FC (NSAF), median	Log2
P07360	C8G	Complement component C8 gamma chain	22.26	0.22	0.24	0.83	1.00	0.00
P09871	C1S	Complement C1s subcomponent	76.63	0.84	0.82	0.92	1.00	0.00
P23142	FBLN1	Fibulin-1	77.16	0.06	0.16	0.94	1.00	0.00
P35542	SAA4	Serum amyloid A-4 protein	14.74	0.86	0.76	0.98	1.00	0.00
Q6UXB8	PI16	Peptidase inhibitor 16	49.44	0.46	0.26	0.95	1.00	0.00
Q8NGG7	OR8A1	Olfactory receptor 8A1	36.36	0.02	0.02	1.00	1.00	0.00
Q969T7	NT5C3B	7-methylguanosine phosphate-specific 5'-nucleotidase	34.37	0.28	0.28	0.82	1.00	0.00
Q96C24	SYTL4	Synaptotagmin-like protein 4	75.98	0.1	0.02	1.00	1.00	0.00
Q9BV73	CEP250	Centrosome-associated protein CEP250	280.97	0.02	0.02	1.00	1.00	0.00
P00751	CFB	Complement factor B	85.48	0.98	0.98	0.96	1.03	0.04
P19652	ORM2	Alpha-1-acid glycoprotein 2	23.59	1	0.98	0.98	1.04	0.05
P00739	HPR	Haptoglobin-related protein	39.00	0.34	0.5	1.05	1.04	0.06
A0A075B6S5	IGKV1-27	Immunoglobulin kappa variable 1-27	12.70	0.02	0.06	1.00	1.06	0.08
P04196	HRG	Histidine-rich glycoprotein	59.54	0.98	0.94	1.08	1.07	0.09
P25311	AZGP1	Zinc-alpha-2-glycoprotein	34.24	0.98	0.94	1.12	1.08	0.11
P13645	KRT10	Keratin, type I cytoskeletal 10	58.79	0.02	0.04	1.08	1.08	0.12
P80748	IGLV3-21	Immunoglobulin lambda variable 3-21	12.44	0.54	0.24	0.99	1.08	0.12
P01860	IGHG3	Immunoglobulin heavy constant gamma 3	41.26	0.98	0.84	1.09	1.11	0.15
A0A0B4J1Y9	IGHV3-72	Immunoglobulin heavy variable 3-72	13.19	0.02	0.16	1.15	1.15	0.20
P01701	IGLV1-51	Immunoglobulin lambda variable 1-51	12.24	0.08	0.12	0.96	1.17	0.22
H3BNL8	ARMH2	Armadillo-like helical domain-containing protein 2	26.53	0.34	0.32	1.08	1.18	0.23
Q06033	ITIH3	Inter-alpha-trypsin inhibitor heavy chain H3	99.79	0.18	0.12	0.94	1.20	0.26
P04003	C4BPA	C4b-binding protein alpha chain	66.99	1	0.98	1.21	1.20	0.26
P05156	CFI	Complement factor I	65.71	0.96	0.82	1.15	1.23	0.30
P01824	IGHV4-39	Immunoglobulin heavy variable 4-39	13.91	0.12	0.44	1.38	1.25	0.32
P05160	F13B	Coagulation factor XIII B chain	75.46	0.04	0.18	0.91	1.25	0.32
P68871	HBB	Hemoglobin subunit beta	15.99	0.96	0.9	1.22	1.33	0.41
P22352	GPX3	Glutathione peroxidase 3	25.54	0.72	0.5	0.96	1.33	0.42
Q96IY4	CPB2	Carboxypeptidase B2	48.39	0.02	0.36	1.29	1.39	0.47
P04275	VWF	von Willebrand factor	309.06	0.16	0.12	1.52	1.50	0.58
P22891	PROZ	Vitamin K-dependent protein Z	44.71	0.16	0.1	1.15	1.50	0.58
P26927	MST1	Hepatocyte growth factor-like protein	80.27	0.4	0.18	1.28	1.50	0.58
P49327	FASN	Fatty acid synthase S-acetyltransferase	273.25	0.44	0.14	1.33	1.50	0.58
P49908	SELENOP	Selenoprotein P	43.16	0.04	0.04	1.50	1.50	0.58
Q8NEE6	FBXL13	Dynein regulatory complex subunit 6	83.87	0.14	0.3	1.42	1.50	0.58
O75882	ATRN	Attractin	158.43	0.74	0.52	1.63		
P01602	IGKV1-5	Immunoglobulin kappa variable 1-5	12.77	0.06	0.3	1.66	1.65	0.72
O00151	PDLIM1	PDZ and LIM domain protein 1	36.05	0.02	0.04	2.00	2.00	1.00
Q9NUJ7	PLCXD1	PI-PLC X domain-containing protein 1	36.64	0.02	0.02	2.00	2.00	1.00

Supplementary Table B2. Proteins that did not pass the significance score in the validation cohort of patients with schizophrenia.

Primary gene name	Pathway description	Study cohort, SCZ patients		Validating cohort			
		Fold changes	Adjusted *p*-value	Concentration, mean ± SD, nM/L		Fold changes	Adjusted *p*-value*
				SCZ group	Control group		
GPX3 †	Thyroid hormone synthesis (hsa04918); Arachidonic acid metabolism (hsa00590); Glutathione metabolism (hsa00480); Pathways of neurodegeneration - multiple diseases (hsa05022)	-2.29	5.39E-03	0.026 ± 0.141	0.080 ± 0.144	-1.6	1.14E-01
IGKC †	Immune response-	-0.82	9.07E-07	185.1 ± 79.51	203.8 ± 46.86	-0.14	2.50E-01
C2 †	Complement and coagulation cascades (hsa04610); Corovirus disease - COVID-19 (hsa05171)	1.92	6.31E-04	0.795 ± 0.917	0.568 ± 0.633	0.49	2.31E-01
ATRN †	Leukocyte transendothelial migration (hsa04670);	2.79	5.94E-03	0.244 ± 0.516	0.094 ± 0.224	1.38	1.31E-01

2. Proteins interaction

Selection of n-24 proteins were treated with STRING analysis at a statistical significance of p<0.01. The analysis returned tight interaction of almost complete set of proteins (Supplemental Figure 1B) with local PPI (protein-protein interactions) clustering coefficient of 0.971 (p<1.0e-16).

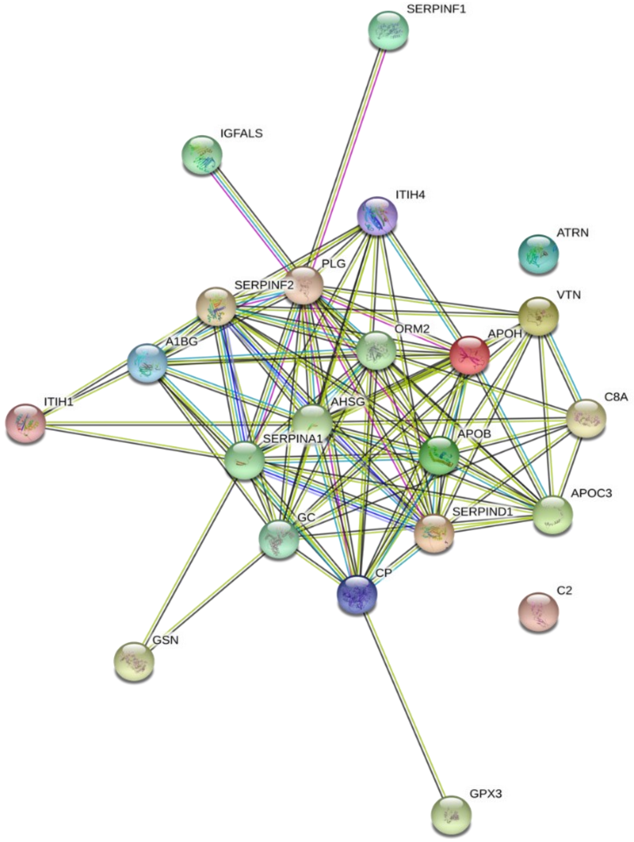

Supplemental Figure 1B: protein-protein interactions network as resulted after treatment the extracted set of n=24 meaningful proteins with STRING. The local clustering coefficient achieved 0.971 with p<1.0e-16 and clustering power of 0.7 (strong).

On the one hand, it was not surprising since all the selected proteins were serum-based markers and may contribute to a vast number of interplaying biological processes. On the other hand, it was amusing since obtaining of such dense core of cluster interactions may evidence about not-occasional extraction of proteins as a result of analysis and, consequently, depletion of mostly irrelevant and generally circulating proteins.

It is hard to attribute the exact molecular functions and biological processes where the designed proteins set implicated in, because most of these serum markers are actors in numerous interplaying cascades and pathways. Most prominent and significant of them are designated in the Main Paper (see section “Results” and section “Discussion where appropriate). However, it seems on the surface that there are many participants in lipids transport and transformation including routine transformation of cholesterol to steroidogenesis, regulation of immune response and clearance of insulin etc. Apart obvious regulation of inflammation and response to stress, there were also related transport of retinoic acid, steroids and primary amines, including those which feed a source of dopamine and serotonin synthesis. Complete description of Biological Processes, Molecular Functions, Reaction Pathways and Subcellular Localizations were aggregated and listed below in tables (after “Appendix B References” section).

Appendix B References:

[1] Zybailov B, Coleman MK, Florens L, Washburn MP. Correlation of relative abundance ratios derived from peptide ion chromatograms and spectrum counting for quantitative proteomic analysis using stable isotope labeling. Anal Chem. 2005;77(19):6218-6224. doi:10.1021/ac050846r
[2] Zybailov B, Mosley AL, Sardiu ME, Coleman MK, Florens L, Washburn MP. Statistical analysis of membrane proteome expression changes in Saccharomyces cerevisiae. J Proteome Res. 2006;5(9):2339-2347. doi:10.1021/pr060161n
[3] Zhang Y, Wen Z, Washburn MP, Florens L. Refinements to label free proteome quantitation: how to deal with peptides shared by multiple proteins. Anal Chem. 2010;82(6):2272-2281. doi:10.1021/ac9023999

[4] McIlwain S, Mathews M, Bereman MS, Rubel EW, MacCoss MJ, Noble WS. Estimating relative abundances of proteins from shotgun proteomics data. BMC Bioinformatics. 2012;13:308. doi:10.1186/1471-2105-13-308
