# Supplementary material for: Consolidation of metabolomic, proteomic, and GWAS data in connective model of schizophrenia
Source: Sci Rep. 2023 Feb 6;13:2139. doi: 10.1038/s41598-023-29117-7 (PMC9901842; doi:10.1038/s41598-023-29117-7)
Supplement: Supplementary file 4 — Supplementary Information 4. [file 41598_2023_29117_MOESM4_ESM.pdf]

Appendix D

Metabolome: list of the most altered metabolites among patients with schizophrenia and healthy donors. This Appendix congregates metabolites irrespective of whether they meet the criteria of statistical significance (p-value). Thence, even statistically insignificant compounds but with substantial fold changes can be met in among listed metabolites

Authors: Arthur T. Kopylov, Alexander A. Stepanov, Tatiana V. Butkova, Kristina A. Malsagova, Natalia V. Zakharova, Georgy P. Kostyuk, Artem U. Elmuratov, Anna A. Kaysheva

Connective model of schizophrenia: a roadmap in maze of metabolomic, proteomic and GWAS data

Arthur T. Kopylov, Alexander A. Stepanov, Tatiana V. Butkova, Kristina A. Malsagova, Natalia V. Zakharova, Georgy P. Kostyuk, Artem U. Elmuratov, Anna A. Kaysheva

Appendix D: Metabolome

Content

1. Metabolites meaningful in pathogenesis of schizophrenia 1

1. Metabolites meaningful in pathogenesis of schizophrenia

Metabolomics data are typically vague and redundant which especially characterized for the untargeted mass spectrometry approaches. Thence, our initial data contained a tremendous peak-lists which represented by more than a thousand primary identifications in each studied group. Obviously, the majority of these identification are false and irrelevant due to aforementioned reasons of wide redundancy of metabolomic data. We surmised about enhanced influence of drug and drug-like compounds putatively circulated in the analyzed serum samples as well as native compound generally represented due to, for example, dietary preference. Therefore, we accomplished stepwise percolation of initial lists of compounds including statistical analysis (see Supplemental Methods and “Results” section in the Main Paper) and manual curation. In summary, we eliminated more than 200 drug-like metabolites and about 100 food compounds.

The obtained shortened lists were further statistically treated and showed that nearly 30% of the rest compounds are shadowed by isomers (which typical for abundant lipids and their derivatives). Since we could not attribute the exact structure match in such cases, these compounds were also excluded from the consideration. Finally, 142 metabolites were determined as overlapped between patients with schizophrenia and healthy donors but only 24 were elected as meaningful as a result of PCA analysis. The upshot is 18 metabolic determinants which can be considered as statistically significant and related to schizophrenia onset and progression according the literature overview (see “Discussion” section in the Main Paper).

Expectedly, the prevalence of steroids, especially, androgenic steroids, and amines transformation products with incline to tyrosine transformation (including catecholamines) was observed in the final list of metabolic compounds (see Table 3 in the Main Paper) with fold-changes more than 2 and less than 0.5 compared to the control group baseline (at adjusted p-value cut-off $p<0.05$ by Mann-Whitney U-test). Although there were 6 compounds that did not pass the statistical significance test (p-value was more than 0.05), we still hold them in the final list due to confidently observed substantial fold-changes in patients with schizophrenia which putatively awards important biological properties to such compounds in the pathogenesis of the disease (see footnotes to Table 3 in the Main Paper). The finally elected metabolites (n=18) and related metabolites were extracted from the data and incorporated in the reconstructed chain of molecular events crucial for schizophrenia pathogenesis where proteome and metabolome layers became complementary.

Supplementary Table 1D. List of metabolites observable as most significantly fluctuated amongst patients with schizophrenia and healthy donors. These metabolites were accumulated from the list of shared metabolites and may contain statistically insignificant elements, however they still accommodated as compounds exhibiting pronounced biological significance and relevance to the pathology under consideration.

Compound name	Empirical formula	LIPIDS MAP ID	HMDB ID	KEGG ID	Fold-changes, arb. Units	Log(2)FC, arb. Units	p-value (U-test)	Log(10) p-value
Serotonin	C10H12N2O		HMDB00259	C00780	0.1042	-3.2622	0.0010	3.0010
C16 Sphinganine	C16H35NO2	LMSP01040001			0.1472	-2.7640	0.8500	0.0706
thyrotropin releasing hormone	C16H22N6O4				0.2033	-2.2982	0.0001	3.9878
3-O-Sulfogalactosylceramide	C48H93NO12S		HMDB00024	C06125	0.2712	-1.8824	0.0002	3.6212
Estrone	C18H22O2	LMST02010004	HMDB00145	C00468	0.2748	-1.8633	0.0068	2.1655
24-oxocholesterol	C27H44O2	LMST01010133			0.3071	-1.7032	0.0569	1.2446
Metanephrine	C10H15NO3		HMDB04063	C05588	0.3638	-1.4588	0.6017	0.2207
Androsterone glucuronide	C25H38O8		HMDB02829	C11135	0.3924	-1.3495		
Thyroxine	C15H11I4NO4		HMDB00248	C08212	0.4740	-1.0769	0.0479	1.3198
11-Hydroxyandrosterone	C19H30O3		HMDB02984	C14606	0.6867	-0.5423	0.2523	0.5982
Cholyltaurine	C26H45NO7S				0.7257	-0.4626		
L-3,5-Diiodotyrosine	C9H9I2NO3			C01060	0.7857	-0.3480	0.0003	3.4710
Cholylglycine	C26H43NO6				0.9094	-0.1370	0.4634	0.3340
L-Histidinol phosphate	C6H12N3O4P			C01100	0.9231	-0.1154	0.8924	0.0494
Cholesterol	C27H46O		HMDB00067	C00187	0.9323	-0.1012	0.7580	0.1203
Pantothenic Acid	C9H17NO5				0.9382	-0.0920	0.9530	0.0209
Testosterone	C19H28O2		HMDB00234	C00535	0.9572	-0.0631	0.1069	0.9709
Epinephrine (adrenaline)	C9H13NO3			C00547	0.9638	-0.0532	0.3392	0.4696
16alpha,17beta-Estriol 3-(beta-D-glucuronide)	C24H32O9	LMST05010017		C11288	1.0110	0.0158		
Tyramine	C8H11NO		HMDB00306	C00483	1.0757	0.1053	0.1372	0.8627
17beta-Estradiol 3-(beta-D-glucuronide)	C24H32O8	LMST05010007		C05503	1.1260	0.1713	0.0594	1.2259
Cholest-5-ene	C27H46		HMDB00941	C05416	1.1389	0.1876		
19-Norandrosterone	C18H28O2				1.2627	0.3365	0.0964	1.0160
Cholesterol sulfate	C27H46O4S		HMDB00653		1.5908	0.6698	0.0028	2.5539
Sphinganine 1-phosphate	C18H40NO5P		HMDB01383	C01120	1.7675	0.8217	0.2227	0.6523
16-a-Hydroxypregnenolone	C21H32O3		HMDB00315	C06390	1.8289	0.8709	0.0018	2.7337
Tyrosine	C9H11NO3		HMDB00158	C00082	1.9049	0.9297	0.7430	0.1290
Dihydrotestosterone	C19H30O2	LMST02020042	HMDB02961	C03917	2.0519	1.0370	0.0021	2.6746
Androstenedione	C19H26O2		HMDB00053	C00280	2.4858	1.3137	0.0281	1.5520
Norepinephrine (noradrenaline)	C8H11NO3			C00547	2.8296	1.5006		
Dopamine	C8H11NO2		HMDB00073	C03758	2.9042	1.5381	0.1506	0.8223
Testosterone sulfate	C19H28O5S		HMDB02833	C00535	3.2326	1.6927	0.4685	0.3293
Testosterone glucuronide	C25H36O8		HMDB03193	C11134	4.8228	2.2699	0.6510	0.1864
Vitamin D3 (7-dehydrocholesterol)	C27H44O			C05443	5.4168	2.4374	0.0472	1.3263

Compound name	Empirical formula	LIPIDS MAP ID	HMDB ID	KEGG ID	Fold-changes, arb. Units	Log(2)FC, arb. Units	p-value (U-test)	Log(10) p-value
Sphingosine	C18H37NO2		HMDB00252	C00319	8.9143	3.1561	0.5556	0.2552
Androsterone sulfate	C19H30O5S		HMDB02759	C04555	12.2352	3.6130	0.0149	1.8267
Dopaquinone	C9H9NO4				20.0365	4.3246		
Androstanediol	C19H32O2		HMDB00495	C07632	66.3970	6.0530		

Supplementary Table 2D. List of metabolites that did not pass the significance score in the validating cohort but were considerable in the study cohort among patient with schizophrenia.

Name of metabolite	Pathway description	Main associated identifiers		Study cohort		Validating cohort	
		KEGG ID	HMDB ID	Fold changes	Adjusted *p*-value	Fold changes	Adjusted *p*-value †
Metanephrine ‡	Tyrosine metabolism (hsa00350)	C05588	HMDB04063	-1.46	6.02E-01	-1.22	0.356
Norepinephrine ‡	Neuroactive ligand-receptor interaction (hsa04080); cAMP signaling pathway (hsa04024); Synaptic vesicle cycle (hsao4721); Chemical carcinogenesis - receptor activation (hsa05207)	C00547	-	1.5	2.37E-01	-	-
Dopamine ‡	Neuroactive ligand-receptor interaction (hsa04080); cAMP signaling pathway (hsa04024); Synaptic vesicle cycle (hsao4721); Chemical carcinogenesis - receptor activation (hsa05207); Prolactin signaling pathway (hsa04917); Tyrosine metabolism (hsa00350); Adrenergic signaling in cardiomyocytes (hsa04261); Regulation of lipolysis in adipocytes (hsa04923); Renin secretion (hsa04924); Salivary secretion (hsa04970); Thermogenesis	C03758	HMDB00073	1.54	1.51E-01	1.87	0.883

Name of metabolite	Pathway description	Main associated identifiers		Study cohort		Validating cohort	
		KEGG ID	HMDB ID	Fold changes	Adjusted *p*-value	Fold changes	Adjusted *p*-value †
	(hsa04714); Vascular smooth muscle contraction (hsa04270)						
Sphingosine ‡	Sphingolipid metabolism (hsa00600); Apoptosis (hsa04210); Necroptosis (hsa04217); Sphingolipid signaling pathway (hsa04071)	C00319	HMDB00252	3.16	5.56E-01	-0.57	0.316
Dopaquinone ‡	Tyrosine metabolism (hsa00350); Betaine biosynthesis (hsa00965);	C00822	HMDB0001229	4.32	1.03E-01	2.58	0.003
Androstanediol ‡	Steroid hormone biosynthesis (hsa00140); Ovarian steroidogenesis (hsa04913)	C07632	HMDB00495	6.05	1.11E-1	3.39	0.071
