# Supplementary material for: Consolidation of metabolomic, proteomic, and GWAS data in connective model of schizophrenia
Source: Sci Rep. 2023 Feb 6;13:2139. doi: 10.1038/s41598-023-29117-7 (PMC9901842; doi:10.1038/s41598-023-29117-7)
Supplement: Supplementary file 5 — Supplementary Information 5. [file 41598_2023_29117_MOESM5_ESM.pdf]

Appendix E

GWAS: commentaries to the genome-wide associated study of schizophrenia (48 cases and 183 controls). Through the meta-analysis we identified 52 associated loci closets to the significance threshold cut-off. The GWAS data in integration with the proteomic and metabolomic data within this study provide its own piece of input of insights in the biology of schizophrenia.

Authors: Arthur T. Kopylov, Alexander A. Stepanov, Tatiana V. Butkova, Kristina A. Malsagova, Natalia V. Zakharova, Georgy P. Kostyuk, Artem U. Elmuratov, Anna A. Kaysheva

Connective model of schizophrenia: a roadmap in maze of metabolomic, proteomic and GWAS data

Arthur T. Kopylov, Alexander A. Stepanov, Tatiana V. Butkova, Kristina A. Malsagova, Natalia V. Zakharova, Georgy P. Kostyuk, Artem U. Elmuratov, Anna A. Kaysheva

Appendix E: GWAS

Content

GWAS analysis of blood samples from schizophrenic patients and healthy volunteers 1

Determination of population uniformity 2

Manhattan plot 3

GWAS analysis of blood samples from schizophrenic patients and healthy volunteers

We collected blood samples from patients with treatment-resistant schizophrenia in Russian Federation. Following the national ethics regulations and in line with the local ethical committee of Alexeev N.A. 1st Clinics of Mental Health, we ascertained anonymous aliquots of the blood samples collected as a part of this study. In addition, we included a more conventional cohort of patients with schizophrenia who also we being for inpatient at the 1st Clinics of Mental Health. We have to note that all these samples are intrinsically related to the research requirements.

All patients enrolled for the study were acquired clinical and consensus diagnosis of schizophrenia. Among participant with treatment-resistant schizophrenia we selected those cases who confirmed the matched diagnosis provided when a participant was started medical treatment. Then we compared a clinical diagnosis with consensus research DSM-5 diagnosis. Following the consensus research diagnosis, 49 subjects from the study cohort were met the criteria of DSM-5 for schizophrenia.

A quality control allowing only 2% of missing SNPs was performed separately in each individual dataset using PLINK version 1.90b6.7 following the standard procedures described in [1]. We guided the following case-control quality control selection principles [2]:

- *case and control are taken from the same population;*
- *case is selected, erroneous cases will significantly reduce the statistical power of the study;*
- *genetic and epidemiological data of cases and controls are collected in the same way;*
- *the selected controls should be at risk of developing a phenotype, e.g. women should not be used when studying male phenotypes.*

Genotyping data included 652,297 markers for 248 individuals. The analyzed 248 samples consisted of 48 cases and 200 controls. Filters were applied to increase the accuracy (Table 1E). After filtration, 455,922 markers and 231 individuals remained, of which 48 cases and 183 controls.

Table 1E. The following filters were applied to the selection of individuals for genotyping.

Parameter	Threshold value	Exclusion	Description
–mind	0.1	0	Excluding Samples with Low Call Rate
–geno	0.05	26253	Excluding SNP with Low Call Rate
–hwe	0.001	20418	Excluding markers that fail the Hardy-Weinberg test
–maf	0.01	149704	Excluding SNP with Low MAF
–me	1 1		Zeroing genotypes containing Mendelian errors

Determination of population uniformity

Quality control and further association study was performed using plink v1.90b6.7 tool [https://www.cog-genomics.org/plink/1.9/] A principal component analysis (PCA) of common variant (MAF higher than 5%) was carried out to obtain a general summary of population structure, using smart-PCA version 13.0.50 of EIGENSOFT toolset [3]. A comparison performed by PCA, showed that population is homogenous enough but still there was a small fraction of cases with no overlapping controls (Figure 1E).

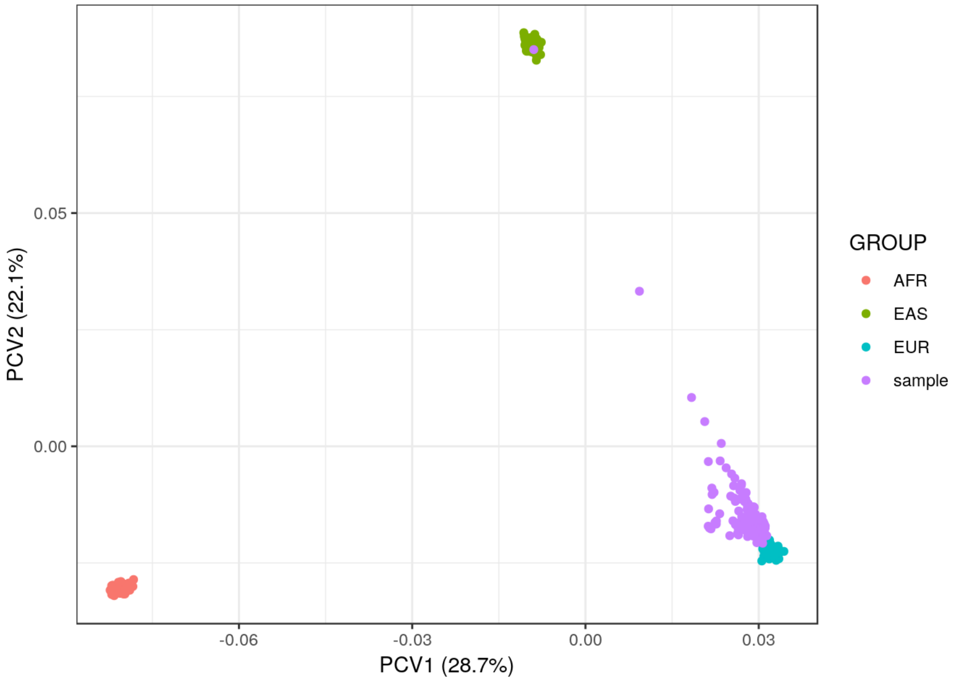

Figure 1D: Population structure of the complete study dataset selected for the GWAS. PCA plot shows cases and controls, notice the moderate spread in the cases.

A plot of the first two principal components demonstrated the existence of cases with similar to genetic ancestor to non-European individuals, probably from the East Asian [4]. In order to use only cases with matching control samples and to ameliorate population stratification in the GWAS analysis, all cases that did not fall into the area delimited by the mean and three standard deviations of the two first principal components of the control samples were excluded from further analyses (Figure 2E).

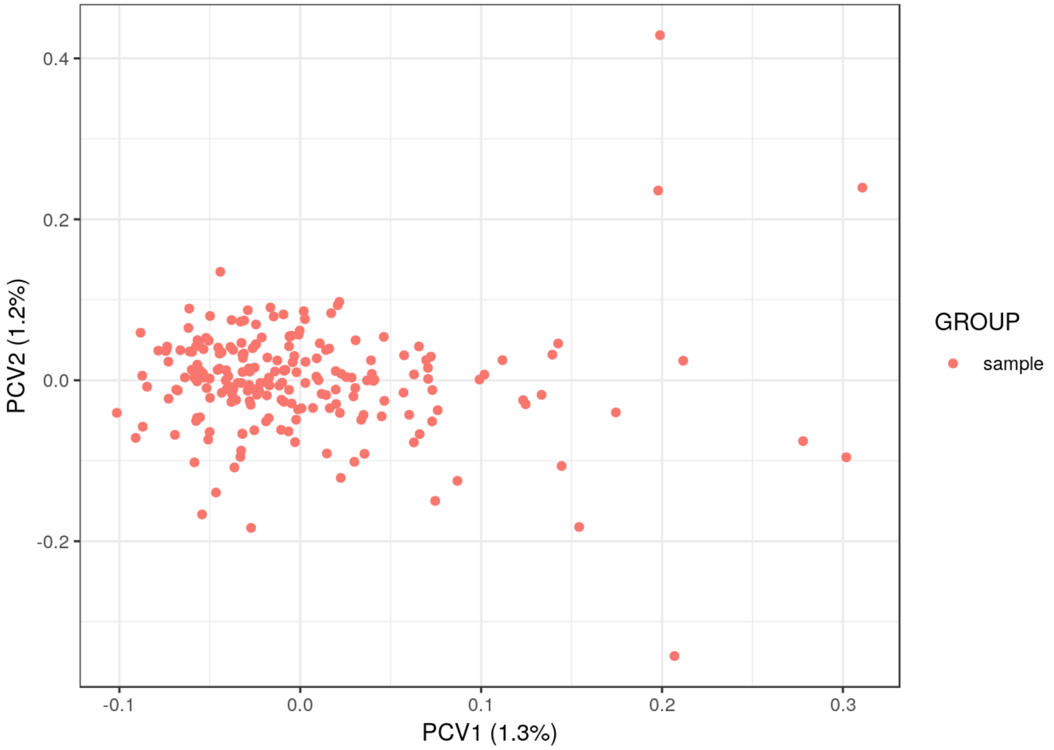

Figure 2E: Population structure of the subset selected for the GWAS after eliminating of outliers. PCA plot shows cases and controls, notice the profiles are almost completely overlapping.

After repeating the PCA analysis, only the matching individuals with no any outliers could be detected in the first two principal components (**Figure 2E**).

Manhattan plot

The GWAS of patients with schizophrenia was performed using logistic regression with imputation probabilities adjusted for PCA covariates, chosen as nominally significant with $p<0.05$ in the employed logistic regression **[5]**. To avoid overloading the GWAS by adding too many covariates to the regression model, only the first 10 principal components were considered and tested for inclusion. The final set of covariates included the first five principal components as recommended for most GWAS approaches **[6]**. The Manhattan plot is shown in **Figure 3E**.

The analysis used a soft threshold for MAF. We used the filter C_U≥10 and C_A≥10. Analysis of GWAS results excluding the related and overlapping samples did not identify genome-wide significant polymorphisms ($p=10^{-5}$) that would distinguish the studied cohorts. Hence, polymorphisms closest to the cut-off threshold (top-100) were selected, 52 of which were localized in protein-coding regions and considered as auxiliary for the reconstructing of vulnerable processes in cross-layer molecular analysis (**Table 2E**).

Arthur T. Kopylov, Alexander A. Stepanov, Tatiana V. Butkova, Kristina A. Malsagova, Natalia V. Zakharova, Georgy P. Kostyuk, Artem U. Elmuratov, Anna A. Kaysheva

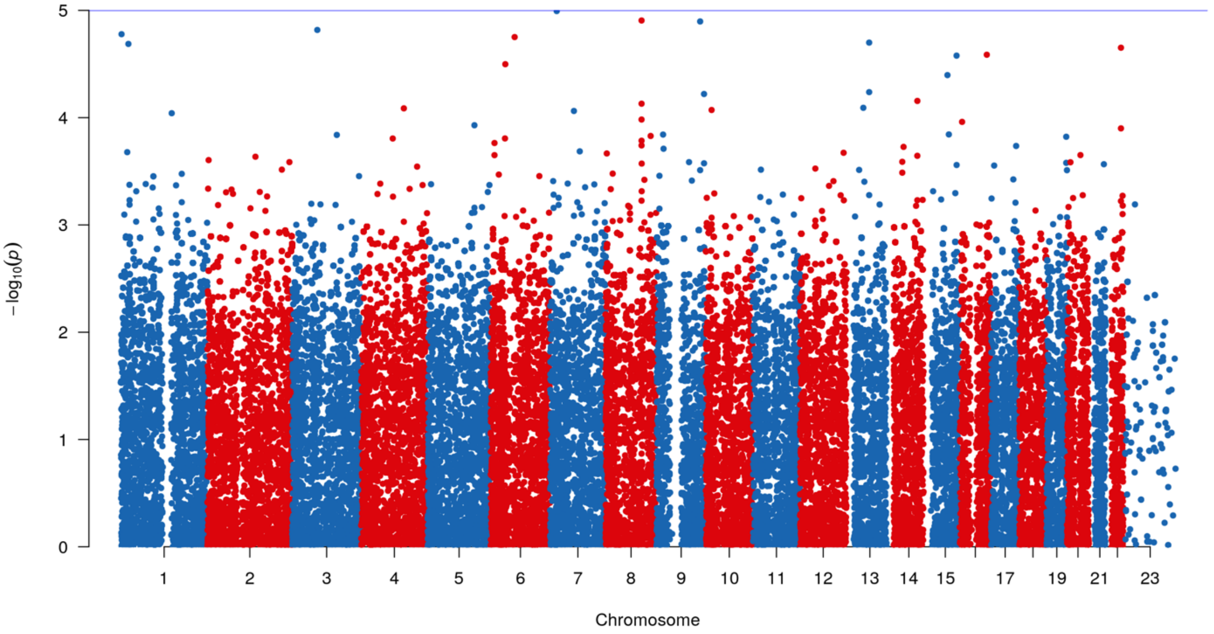

Figure 3E: The Manhattan plot of schizophrenia GWAS study. Associations are shown from the meta-analysis of the study cohort and an independent 1000 Genome Phase dataset (455,922 markers and 231 individuals remained, of which 48 cases and 183 controls). The blue horizontal line on the top of the graph indicates the genome-wide statistical significance threshold (p=10^{-5}).

At least 20 biological processes were associated with the identified polymorphisms that were coated on the protein and metabolite layers (including hsa04975, hsa04977, hsa04979, hsa00590, hsa05203, hsa04080 etc.; and hsa05034, hsa04024, hsa04080, hsa04726, hsa04742, hsa04976 etc. in proteome and metabolome layers, correspondingly).

Table 2E: Polymorphisms localized in protein-coding regions and selected among top-100 SNPs closests to the significance (p=10^{-5}) cut-off threshold.

ID	Gene	Minor allele	Major allele	Control allele frequency	Numder of control samples with matched SNPs	Case allele frequency
rs6959501	HDAC9	C	T	0.0389	16	0.1667
rs8046	PTGS1	G	A	0.0389	16	0.1667
rs9863682	LINC00870	T	C	0.0273	15	0.1563
rs4707976	LOC101928280<br>RP11-406O16.1	A	G	0.0327	80	0.8333
rs2071883	PNPLA5	A	G	0.0776	22	0.2292
rs58877531	VAT1L	A	G	0.0298	16	0.1667
rs112753914	CHD2	T	C	0.0184	12	0.1250
rs12527133	TRERF1	C	T	0.0311	15	0.1563

Arthur T. Kopylov, Alexander A. Stepanov, Tatiana V. Butkova, Kristina A. Malsagova, Natalia V. Zakharova, Georgy P. Kostyuk, Artem U. Elmuratov, Anna A. Kaysheva

ID	Gene	Minor allele	Major allele	Control allele frequency	Numder of control samples with matched SNPs	Case allele frequency
rs60428975	SMAD3	C	T	0.0455	19	0.1979
rs28515121	ADAMTS13	A	G	0.4841	22	0.2292
rs11254275	CUBN	G	A	0.2654	45	0.4688
rs116843707	RBFOX1	T	C	0.0179	13	0.1354
rs17784142	THSD4	T	C	0.5419	31	0.3229
rs1433819	IMMP2L	A	G	0.0389	16	0.1667
rs1735537	EEFSEC	C	T	0.2232	40	0.4167
rs75576234	ZNF444	T	G	0.0208	12	0.1250
rs9348837	NEDD9	A	G	0.1000	26	0.2708
rs2435279	LINC00972	A	G	0.0179	85	0.8854
rs2230705	ALDH4A1	C	G	0.2378	55	0.5729
rs61941687	SCARB1	T	C	0.2195	39	0.4063
rs2449173	CSMD1	G	A	0.0387	80	0.8333
rs6908326	NEDD9	A	G	0.1786	35	0.3646
rs1897419	THSD7B	A	G	0.1946	37	0.3854
rs10169899	TRAPPC12	G	A	0.1168	28	0.2917
rs28548527	LINC01501	C	T	0.0240	12	0.1250
rs60577234	ZNF444	T	C	0.0180	11	0.1146
rs3124768	ADAMTS13	G	A	0.3855	55	0.5729
rs56978822	NTN1	A	C	0.0298	12	0.1250
rs9574707	DCLK1	A	G	0.1265	29	0.3021
rs10306166	PTGS1	A	G	0.0119	9	0.0938
rs2445858	ZNF274	G	A	0.4018	75	0.7813
rs2179158	TNR	C	T	0.1933	37	0.3854
rs10977029	PTPRD	T	C	0.4222	20	0.2083
rs57580563	FGF12	A	G	0.0357	13	0.1354
rs12405554	BCAR3	C	T	0.0689	19	0.1979
rs12410101	BCAR3	T	C	0.0689	19	0.1979
rs11079590	CEP112	T	C	0.2844	45	0.4688
rs118125840	GABBR2	T	C	0.0119	9	0.0938
rs79358863	LOC105369921	C	A	0.0210	10	0.1042
rs638908	DLEU7	A	G	0.3720	52	0.5417
rs79700936	NEGR1	T	C	0.0089	8	0.0833
rs2299542	GRM8	G	A	0.4345	71	0.7396
rs1043879	GRM8	C	T	0.2143	38	0.3958
rs10908703	FCER1A	A	G	0.0723	19	0.1979
rs117406702	ZAN	A	G	0.0208	10	0.1042
rs284169	TGFBR3	A	G	0.4488	62	0.6458
rs13034507	MYT1L	T	G	0.3303	51	0.5313
rs10099062	SGCZ	T	C	0.0298	12	0.1250
rs61185885	GKN1	G	A	0.0357	13	0.1354
rs12902607	LOC100128714	C	T	0.4792	68	0.7083
rs78213015	TMEM53	A	G	0.0208	10	0.1042
rs16826885	LYPD6	A	G	0.1310	27	0.2813

Appendix E References:

[1] Anderson, C.A. et al. Data quality control in genetic case-control association studies. Nat. Protocols 5, 1564-1573 (2010).
[2] Pearson TA, Manolio TA. How to Interpret a Genome-wide Association Study. JAMA. 2008; 299(11): 1335-1344.
[3] Patterson, N.J., Price, A.L. & Reich, D. Population structure and eigenanalysis. PLoS Genetics 2, e190 (2006).
[4] Tian, C., Gregersen, P.K. & Seldin, M.F. Accounting for ancestry: population substructure and genome-wide association studies. Human Molecular Genetics 17, R143-R150 (2008)
[5] Peloso GM, Lunetta KL. Choice of population structure informative principal components for adjustment in a case-control study. BMC Genet. 2011;12:64. Published 2011 Jul 19. doi:10.1186/1471-2156-12-64
[6] Tucker G, Price AL, Berger B. Improving the power of GWAS and avoiding confounding from population stratification with PC-Select. Genetics. 2014;197(3):1045-1049. doi:10.1534/genetics.114.164285
